# Network Modeling Reveals Prevalent Negative Regulatory Relationships between Signaling Sectors in Arabidopsis Immune Signaling

**DOI:** 10.1371/journal.ppat.1001011

**Published:** 2010-07-22

**Authors:** Masanao Sato, Kenichi Tsuda, Lin Wang, John Coller, Yuichiro Watanabe, Jane Glazebrook, Fumiaki Katagiri

**Affiliations:** 1 Department of Plant Biology, Microbial and Plant Genomics Institute, University of Minnesota, St. Paul, Minnesota, United States of America; 2 Department of Life Sciences, Graduate School of Arts and Sciences, The University of Tokyo, Meguro-ku, Tokyo, Japan; 3 Stanford Functional Genomics Facility, Stanford, California, United States of America; Michigan State University, United States of America

## Abstract

Biological signaling processes may be mediated by complex networks in which network components and network sectors interact with each other in complex ways. Studies of complex networks benefit from approaches in which the roles of individual components are considered in the context of the network. The plant immune signaling network, which controls inducible responses to pathogen attack, is such a complex network. We studied the Arabidopsis immune signaling network upon challenge with a strain of the bacterial pathogen *Pseudomonas syringae* expressing the effector protein AvrRpt2 (*Pto* DC3000 AvrRpt2). This bacterial strain feeds multiple inputs into the signaling network, allowing many parts of the network to be activated at once. mRNA profiles for 571 immune response genes of 22 Arabidopsis immunity mutants and wild type were collected 6 hours after inoculation with *Pto* DC3000 AvrRpt2. The mRNA profiles were analyzed as detailed descriptions of changes in the network state resulting from the genetic perturbations. Regulatory relationships among the genes corresponding to the mutations were inferred by recursively applying a non-linear dimensionality reduction procedure to the mRNA profile data. The resulting static network model accurately predicted 23 of 25 regulatory relationships reported in the literature, suggesting that predictions of novel regulatory relationships are also accurate. The network model revealed two striking features: (i) the components of the network are highly interconnected; and (ii) negative regulatory relationships are common between signaling sectors. Complex regulatory relationships, including a novel negative regulatory relationship between the early microbe-associated molecular pattern-triggered signaling sectors and the salicylic acid sector, were further validated. We propose that prevalent negative regulatory relationships among the signaling sectors make the plant immune signaling network a “sector-switching” network, which effectively balances two apparently conflicting demands, robustness against pathogenic perturbations and moderation of negative impacts of immune responses on plant fitness.

## Author Summary

When a plant detects pathogen attack, this information is conveyed through a molecular signaling network to turn on a large variety of immune responses. We investigated how this plant immune signaling network was organized using the model plant Arabidopsis. Wild type and mutant plants with defects in immune signaling were challenged with a pathogen. Then, expression levels of many genes were measured using microarrays. Detailed analysis of the mutation effects on gene expression allowed us to build a signaling network model composed of the genes corresponding to the mutations. This model predicted that the network components are highly interconnected and that it is very common for network components that mediate different signaling events to inhibit each other. The prevalent signaling inhibitions in the network suggest that only part of the signaling network is usually used but that if this part is attacked by pathogens, other parts kick in and back up the function of the attacked part. We speculate that plant immune signaling is highly tolerant to pathogen attack due to this backup mechanism. We also speculate use of only part of the network at any one time helps minimize negative impacts of the immune response on plant fitness.

## Introduction

To understand the regulation of a particular biological process, it is important to elucidate what structural features of the signaling network regulating the process govern the behavior of the signaling network as a whole [1], [2]. With a complex signaling network, in which components are highly interconnected, this is a challenging task. One problem is that the function of a sector of the network can be compensated by some other sector, and, consequently, functional identification of these sectors by knocking out each of the sectors is difficult. In this example of network compensation, it is assumed that these network sectors are functionally redundant but mechanistically distinct: they are not composed of homologous molecular components. General strategies to efficiently elucidate the structure of a complex signaling network are in demand.

The plant immune signaling network, which regulates defense triggered upon pathogen attack, is such a complex network. Two modes of plant immunity, pattern- and effector-triggered immunity (PTI and ETI) have been characterized in resistance against biotrophic and hemi-biotrophic pathogens [3]. PTI is initiated by recognition of a microbe-associated molecular pattern (MAMP) by the corresponding pattern recognition receptor (PRR), which is typically integrated in the plasma membrane. For example, a fragment of bacterial flagellin, flg22, is a MAMP, and is recognized by the FLS2 receptor-like kinase PRR in Arabidopsis [4]. Pathogens adapted to a particular plant host deliver effectors which interfere with PTI [5]. Countering pathogen effectors, plants have acquired another class of receptors, resistance (R) proteins, that specifically recognize particular effectors, leading to induction of ETI. For example, the Arabidopsis R protein RPS2 indirectly recognizes the *Pseudomonas syringae* effector AvrRpt2 [6], [7].

Although the way pathogen attack is recognized is distinct between PTI and ETI, they are not separate, but rather form an integrated immune system. The intimate relationships between PTI and ETI have been suggested by the facts that many downstream events are shared. For example, in Arabidopsis, MAP kinases 3 and 6 are rapidly and transiently activated in PTI and activated for an extended period in ETI [8]. Reactive oxygen species (ROS) production in PTI is absolutely dependent on the NADPH oxidase RBOHD, and ROS production in ETI is largely dependent on RBOHD [9], [10]. The nitric oxide (NO) signaling sector comprised of NO-associated 1 (NOA1) protein and NIA1 and NIA2 nitrate reductases is also involved in both PTI and ETI [11], [12]. Furthermore, similarities in the PTI and ETI transcriptome responses have been pointed out [13].

The signaling sectors defined by the phytohormones, salicylic acid (SA), jasmonic acid (JA), and ethylene (ET), are important in plant immunity: generally the SA sector for immunity against biotrophic and hemi-biotrophic pathogens and the JA and ET sectors for immunity against necrotrophic pathogens [14], [15], [16]. The iso-chorismate synthase SID2 (ICS1) [17] and the MATE-type transporter EDS5 [18] are required for SA synthesis in response to pathogen attack. NPR1 [19] is a major positive regulator of SA responses. The regulators EDS1 and PAD4 are important for SA accumulation as well as SA-independent signaling functions [20], [21], [22]. The JA sector contains the JAR1 enzyme that produces the JA-Ile conjugate, which is the active form of JA [23], the F-box protein COI1, which responds to JA-Ile by targeting the JAZ transcription repressors for degradation [24], and the JIN1 Myc transcription activator [25]. The metal-ion transporter EIN2 is required for most ET responses [26], and the EIN3 transcription activator positively regulates some ET responses [27]. Other phytohormones, such as abscisic acid, auxin, brassinosteroids, and gibberellins, are also involved in plant immune signaling [28]. Although the phytohormone levels change during PTI and ETI, the specific effects of the phytohormone sectors in PTI and ETI had been considered to be limited or unclear [3], [29], [30].

Recently, we demonstrated that both flg22-triggered PTI (flg22-PTI) and AvrRpt2-triggered ETI (AvrRpt2-ETI) are mostly dependent on the signaling network defined by the SA, JA, ET and PAD4 sectors [31]. Therefore, the signaling machinery is extensively shared between flg22-PTI and AvrRpt2-ETI. A main difference between PTI and ETI appears to reside in how the sectors in the common network interact one another. If this is true, then to further our understanding of the integrated plant immune signaling network, it is important to elucidate the global regulatory relationships among the network components.

One major use of mRNA profiles is as detailed descriptions of biological states, because an mRNA profile data set is a massive phenotypic data set. This use was pioneered by the “compendium” approach, in which mutations and chemicals that cause similar changes in mRNA profiles are hypothesized to be involved in the same biological processes [32]. In our earlier studies, we implemented non-linear dimensionality reduction [33] in combination with graphical representation to reveal multi-dimensional relationships with locally variable dimensionalities among the mRNA profiles [34], [35], [36]. In this way, information about the nature of similarities between mRNA profiles was obtained in addition to the scalar similarities, and novel relationships among Arabidopsis mutants and accessions were discovered.

Here, we report an integrated regulatory relationship model comprised of 22 components including most of the genetically-defined major regulators of immunity in Arabidopsis. The network structure was inferred based on mRNA profiles for 571 immune response genes of Arabidopsis mutants with defects in immune regulatory genes. The mRNA profiles were collected at a single time point six hours post inoculation (hpi) with the bacterial strain *P. syringae* pv. *tomato* DC3000 expressing the effector AvrRpt2 (*Pto* DC3000 AvrRpt2). This strain feeds multiple inputs to the network. The regulatory relationships were inferred by recursively applying a non-linear dimensionality reduction procedure, which allowed detection of many weak relationships. The model correctly predicted 23 out of 25 previously known relationships, suggesting the accuracy of newly predicted relationships. Two features of the network model were readily evident: the network components were highly interconnected; and negative regulatory relationships between signaling sectors were very common. We confirmed the latter point in one case by demonstrating a mutual inhibition between the SA and early MAMP-triggered (EMT) signaling sectors. Based on the prevalent negative regulatory relationships, we propose “sector-switching” as an important property of the plant immune signaling network.

## Results

### The procedure for inferring the regulatory relationships among components of the Arabidopsis immune signaling network

#### mRNA profiling

mRNA profiling was used to collect detailed descriptions of the network state, and the changes in the network state were determined by comparing the mutant mRNA profiles with the wild-type mRNA profile. One advantage of this approach is that regulatory mechanisms defined by the mutations do not have to be regulated at the mRNA level. For example, a biological process that is regulated by the activity of a protein kinase can be studied using the mRNA profile of the protein kinase mutant even when the mRNA level of the protein kinase is not regulated in this process. This is because the mRNA levels of particular genes were not used as proxies for the activities of the gene products, instead, the mRNA profile changes in a mutant plant compared with the wild-type plant were used as the effects caused by the mutation. Another advantage is that the number of genes in the profiles need not be very high: the genes to be profiled only need to cover (almost) all the expression patterns across the mutants used in the study. We previously reported a dedicated custom microarray that accurately monitors the mRNA levels of 571 Arabidopsis genes, which represent mRNA profile patterns across many conditions related to pathogen infections [37]. Use of this small-scale microarray made this project economical even though we used three biological replicates for profiling.

#### Inputs to the network

We collected mRNA profiles of the mutants and the wild type Columbia-0 (Col-0) from leaf tissues after inoculation of *Pto* DC3000 AvrRpt2. The inoculation dose was sufficiently high for most parenchymal cells to have direct contact with the bacteria. As parenchymal cells are predominant in leaf tissues, this biological system is relatively homogenous at the cellular level. The bacterial strain can stimulate multiple signaling pathways: AvrRpt2 triggers RPS2-mediated ETI, which involves SA-mediated signaling and ROS and NO bursts [9], [38], [39], [40]; the phytotoxin coronatine produced by the strain mimics JA-Ile and activates JA-mediated signaling [41]; MAMPs, such as flg22, trigger PTI, whose early responses include MAP kinase 3 and 6 (MPK3/6) activation, ROS and ET bursts, and callose deposition [42], [43], [44], [45]. Thus, this strain feeds inputs into the network from multiple different points, which allows us to probe a large part of the network at once.

#### Perturbations of the network by mutations

Arabidopsis mutants with defects in canonical immune signaling components were used to specifically perturb various points in the signaling network. Table 1 lists the Arabidopsis mutants used in this study, the functions of the corresponding genes, and their signaling sector assignments.

**Table 1:** Arabidopsis mutants used in this study.

Mutant name	Locus	Short description	Assigned signaling sector^a^	Reference
noa1-1	At3g47450	NOA1 (NO Associated 1); GTPase/nitric-oxide synthase	NO	[71]
AtrbohD	At5g47910	RBOHD (Respiratory Burst Oxidase Homologue D); NAD(P)H oxidase	ROS, EMT	[72]
AtrbohF	At1g64060	RBOHF (Respiratory Burst Oxidase Homologue F); NAD(P)H oxidase	ROS	[72]
coi1-1	At2g39940	COI1 (COronatine Insensitive 1); ubiquitin-protein ligase	JA	[73]
dde2-2	At5g42650	DDE2 (Delayed DEhiscence 2)/AOS (Allene Oxide Synthase); allene oxide synthase	JA	[74]
ein2-1	At5g03280	EIN2 (Ethylene INsensitive 2); transporter	ET, EMT	[26]
ein3-1	At3g20770	EIN3 (Ethylene INsensitive 3); transcription factor	ET, EMT	[27]
jar1-1	At2g46370	JAR1 (JAsmonate Resistant 1); jasmonate-amino synthetase; a member of the GH3 family	JA	[23]
jin1-1	At1g32640	JIN1 (Jasmonate INsensitive 1); MYC2; transcription factor	JA	[25]
mpk3	At3g45640	ATMPK3 (Arabidopsis Thaliana Mitogen-Activated Protein Kinase 3); MAP kinase	MPK3/6, EMT	[75]
mpk6-2	At2g43790	ATMPK6 (Arabidopsis Thaliana Mitogen-Activated Protein Kinase 6); MAP kinase	MPK3/6, EMT	[76]
nho1-2	At1g80460	NHO1 (Nonhost resistance to P. s. phaseolicola 1); glycerol kinase	Misc.	[77]
nia2	At1g37130	NIA2 (NItrate reductase Apoprotein2); nitrate reductase	NO	[78]
ndr1-1	At3g20600	NDR1 (Non race-specific Disease Resistance 1); a plasmamembrane protein	R gene	[79]
npr1-1	At1g64280	NPR1 (Nonexpresser of PR genes 1); transcription cofactor	SA	[19]
pad4-1	At3g52430	PAD4 (PhytoAlexin Deficient 4); lipase-like	SA	[21]
pbs2-1	At5g51700	PBS2 (PphB Susceptible 2); a protein with two zinc binding (CHORD) domains	R gene	[80], [81]
pen2-1	At2g44490	PEN2 (PENetration 2); hydrolase, hydrolyzing O-glycosyl compounds/thioglucosidase	Misc.	[82]
pmr4-1	At4g03550	PMR4 (Powdery Mildew Resistant 4); GLUCAN SYNTHASE-LIKE 5; 1,3-beta-glucan synthase	Callose, EMT	[52]
rps2-101C	At4g26090	RPS2 (Resistance to P. Syringae 2); a NB-ARC protein	R gene	[83], [84]
sag101-2	At5g14930	SAG101 (Senescence-Associated Gene 101); lipase-like	Misc.	[85]
sid2-2	At1g74710	SID2 (SALICYLIC ACID INDUCTION DEFICIENT 2); isochorismate synthase 1 (ICS1)	SA	[17]

a They indicate signaling sectors mediating signals of Callose, callose deposition; ET, ethylene; JA, jasmonic acid; MPK3/6, MAP kinases 3/6; Misc., miscellaneous function; NO, nitric oxide; R gene, resistance gene; ROS, reactive oxygen species; SA, salicylic acid; EMT, early MAMP-triggered.

#### Time point

A single time point of six hpi was chosen for cost-effectiveness. The time point was determined based on our previous observations [46]: the number of genes with expression changes was much higher at 6 hpi than 3 hpi; and while the profile at 9 hpi was similar to that at 6 hpi, we reasoned that the earlier profile may contain more relatively early effects of the genetic perturbations.

#### Network inference

The principle used in network inference is that genes whose mutations cause similar effects on mRNA profiles share regulatory relationships: one regulates the other, both similarly regulate the mRNA levels of the same genes, both are regulated by the same regulator, or the relationships are a combination of these. Such regulatory relationships were visualized by links between the vertices corresponding to the mutant genes in a graphical representation of the network: a positive link when the direction of observed mRNA level changes was the same and a negative link when the direction was opposite.

**Figure 1:**
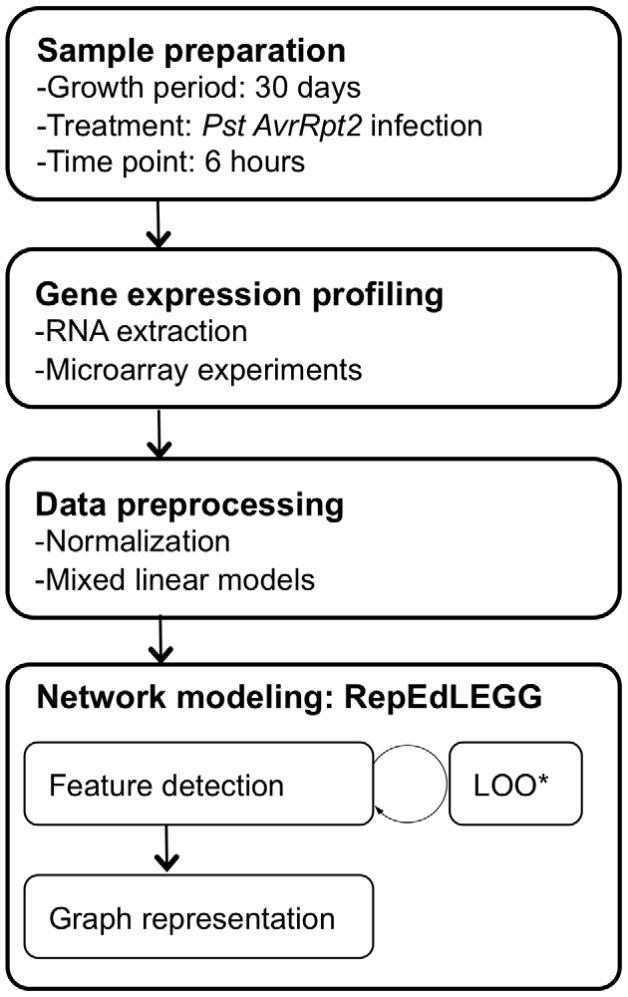
The workflow for network inference.

The mRNA profiles were collected in multiple experiment groups and combined into a single data set using mixed linear models (MATERIALS AND METHODS, Table S1). The overall experimental design regarding the experiment group was not symmetric, and the overlapping genotypes in any particular combination of experiment groups were limited. These features may have introduced some biases in the data set. To compare mutation effects, log_2_-transformed expression values of genes in the wild type mRNA profile were subtracted from log_2_-transformed expression values of genes in each mutant mRNA profile, and the obtained log-transformed mRNA profile change was scaled across the genes, but not centered, to preserve the signs of the values (which is called a difference profile hereafter). Linear dimensionality reduction was applied locally (Locally Linear Embedding, LLE; [33]), so that the same types of mRNA profile changes do not make redundant links. Although the above procedure is in principle the same as used in our previous studies [34], [35], [36], we implemented an additional concept in the current study. In the previous procedure, mutant difference profiles that are local to a particular mutant difference profile are defined based on the global distance in the difference profile space. However, mutants that have a weak regulatory relationship, such as one corresponding to weak cross-talk, may not be detected as their difference profiles may not be located closely in the global space. In the new procedure, named Repetitive Euclidean-distance Locally linear Embedded Graph Generator (RepEdLEGG), the residual from the first round of LLE was subjected to another round of LLE. This recursive application of LLE enabled detection of such weak regulatory relationships (Figure S1). The overall workflow of the network inference procedure is summarized in Figure 1.

### Evaluation of the immune signaling network model using previous information

**Figure 2:**
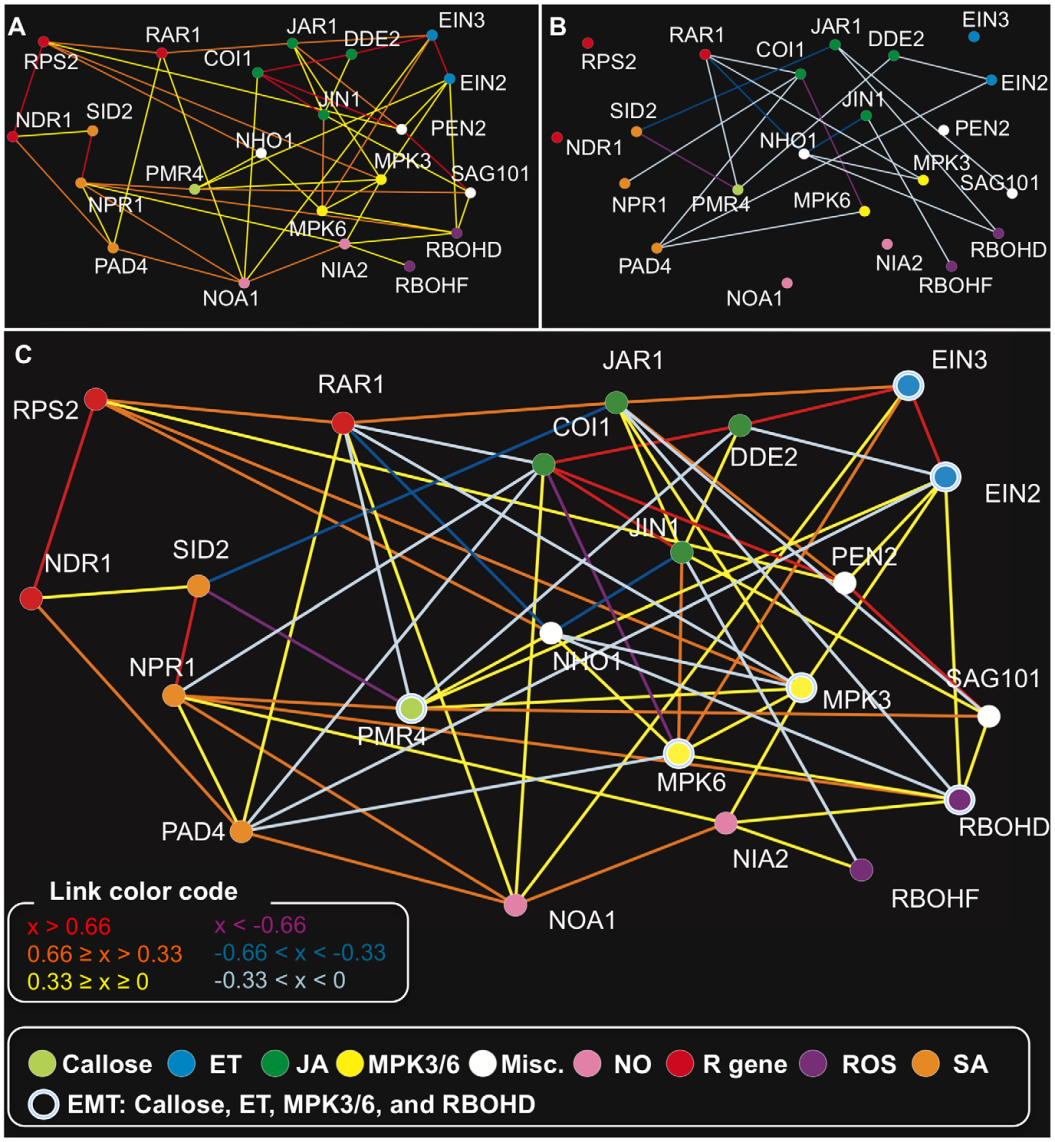
The network model for the genes corresponding to the mutations. The difference profiles of the 22 Arabidopsis mutants at 6 hpi of *Pto* DC3000 AvrRpt2 were analyzed by RepEdLEGG to obtain this network model. Positive (A), negative (B), and both (C) regulatory relationships are graphically represented. See the color codes of the coefficients associated with the links in (C). The color codes for the vertices at the bottom of the figure show the signaling sector assignments for the genes corresponding to the mutations. The links represent the regulatory relationships between the genes. The color codes for the links show the coefficient values obtained in the RepEdLEGG procedure (x). A larger absolute value of x represents a stronger regulatory relationship.

With the above procedure, we obtained a regulatory relationship model for 22 genes corresponding to the mutations with 67 undirected links, which we refer to as our network model (Figure 2). Our network model has a form of an undirected graph since a single time-point data set does not allow inference of the direction of relationships without an additional assumption. Forty-eight and 19 links represented positive and negative regulatory relationships, respectively (Figure 2A and 2B, Figure S2, Table S2). To evaluate the accuracy of the predicted regulatory relationships, the published literature was surveyed for supporting experimental data (Table S3). Twenty-five pairwise regulatory relationships between genes used in this study, that included information about the sign of the relationships, were found in published literature. Our network model correctly predicted 23 out of the 25 known regulatory relationships. One of the relationships not correctly inferred was the JIN1-MPK6 relationship: MPK6 was described as a negative regulator of JIN1 [47] whereas our model predicts a positive relationship between them. The other was that the model did not predict a direct relationship corresponding to negative regulation of SID2 by EIN3, described in Chen *et al.* [48]. However, when JAR1, which was connected positively and negatively with EIN3 and SID2, respectively, was removed from the input data set, the negative regulatory relationship between EIN3 and SID2 was inferred (Table S4). Under our experimental conditions, JA signaling could be strong due to coronatine and may have masked the effect of EIN3, which mediates ET signaling. Note that the known links were established with data from diverse experiments conducted using various Arabidopsis-pathogen interactions, performed by many different research groups. While such studies helped us to select useful mutants for our study, our network model was built based solely on mRNA profile data collected using a single experimental setup with a single time point. This fact demonstrates the richness of information in descriptions of the network state consisting of mRNA profiles and the high efficiency of network inference using mRNA profiles as detailed descriptions of network states. The high accuracy in prediction of previously known regulatory relationships suggests the accuracy of newly predicted regulatory relationships.

The specificities of links can also be examined by removing the data for one mutant from the data set. For instance, a positive link between EIN3 and MPK6 was predicted in the model. This is consistent with the observation in Yoo *et al.* [49] that EIN3 is phosphorylated and activated by MPK6. The direction of this regulatory relationship is from MPK6 to EIN3 but not from EIN3 in the ET sector to MPK6 [49]: in other words, this link is specific to EIN3 but not for the ET sector in general. Therefore, a link between EIN2 in the ET sector and MPK6 should not be made if EIN3 is removed from the model (i.e., if the model is made using the data set with the *ein3* difference profile removed). In the resulting model with EIN3 removed, the link between MPK6 and the other ET signaling component EIN2 was not generated (Figure S3A and A′, Table S5). Thus, the specificity of the biochemical regulation was captured in our network model.

It should be noted that each link may not represent a simple logical relationship. For example, the link between vertices A and B may represent expression changes in one subset of genes profiled and the link between vertices B and C may represent expression changes in a different subset of genes profiled. Therefore, among three vertices a circular link of positive, positive, and negative (e.g., links among MPK3, MPK6, and NHO1) does not necessarily present logical conflicts.

### Characteristics of predicted positive regulatory relationships

As expected, genes assigned to the same signaling sectors were predicted to have positive regulatory relationships except for the ROS sector (Figure 2A). Although RBOHD and RBOHF, the two respiratory burst oxidase homologues, were assigned to the ROS sector, it is known that single *rbohD* and *rbohF* mutants have different pathogen-responsive ROS accumulation and HR cell death phenotypes [9], [10]. Consistently, difference profiles of the two mutants were uncorrelated (uncentered Pearson correlation coefficient between the expression changes from wild type: 0.043). Thus, it is reasonable that no positive link was predicted between the two RBOH genes.

Positive regulatory relationships between signaling sectors were also predicted. Among them, positive regulatory relationships between the NO and SA sectors were of particular interest. In our network model, NOA1 had links with NPR1 and PAD4. Indeed, the *noa1* difference profile had higher correlation with the *pad4* and *npr1* difference profiles than the *nia2* difference profile (uncentered Pearson correlation coefficients of 0.876, 0.831, and 0.714 with the *pad4*, *npr1*, and *nia2* difference profiles, respectively). In the model made without NOA1, NIA2 replaced NOA1 in the links with the two SA sector components, PAD4 and NPR1 (Figure S3B and B′, Table S6). Therefore, the positive regulatory relationships between NOA1 and the SA sector components are not specific to NOA1, but they indicate positive regulatory relationships between the NO and SA sectors in general. On the other hand, the fact that the predicted regulatory relationships between NOA1 and the SA sector are stronger than those between NIA2 and the SA sector is consistent with the observation that NOA1, not NIA1/NIA2, is responsible for SA-induced NO accumulation [50].

### Characteristics of predicted negative regulatory relationships

Negative regulatory relationships are very common between signaling sectors in our network model while negative regulatory relationships within each signaling sector are absent. The NO sector was an exception as it does not have any negative links with other sectors. The JA sector had negative relationships with most of the other signaling sectors tested. The SA sector was negatively linked with PMR4, MPK3/6, and the ET and JA sectors. Prevalent negative regulatory relationships between sectors strongly suggest that a limited number of signaling sectors are highly activated at a given time as the active sectors suppress the other sectors.

### Regulatory relationships between the EMT and the SA sectors

**Figure 3:**
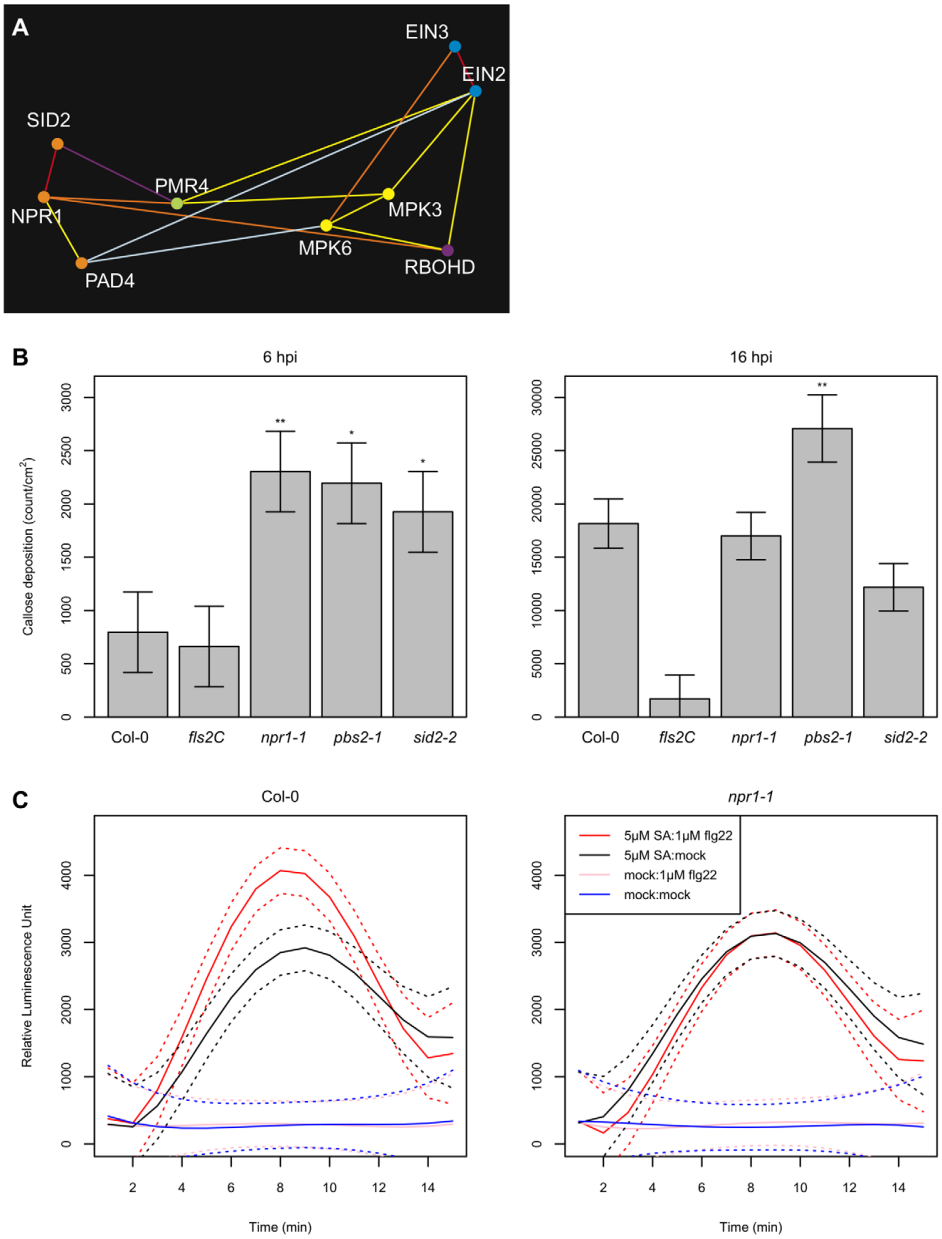
Complex regulatory relationships involving the EMT and SA sectors. (A) A subnetwork of Figure 2C that contains the EMT and SA sectors. (B) flg22-triggered callose deposition was enhanced in SA sector mutants at an early stage. The callose deposition density was measured in wild-type Col-0 and *fls2C*, *npr1-1*, *pbs2-1*, and *sid2-2* mutants at 6 hpt with 1 µM flg22. Ten-day old seedlings in liquid culture were used in this assay. *, *p*<0.05; **, *p*<0.005, compared to the Col-0 value. (C) flg22-triggered ROS generation was enhanced by SA pre-treatment in an NPR1-dependent manner. ROS generation after treatment with either 1 µM flg22 (red, black) or mock (pink, blue) was measured in arbitrary luminescence units in wild-type Col-0 (left) and *npr1-1* (right) over time. Leaf disks prepared from 6-week old plants were pretreated with 5 µM SA (red, pink) or mock (black, blue) 3 hours prior to flg22 treatment. The solid and dashed curves indicate the mean estimates and the 95% confidence intervals, respectively.

Both the EMT and the SA sectors positively contribute to defense against the virulent strain *Pto* DC3000 [43], [45], [51]. Figure 3A illustrates a subnetwork of our network model featuring the EMT and SA sectors. We consider that RBOHD, PMR4, MPK3/6, and the ET sector comprise the EMT sectors because RBOHD-dependent ROS production [10], PMR4-dependent callose deposition [52], MPK3/6 activation [43], and ET accumulation [44] are early MAMP responses. Note that although we designate them as the EMT sectors, RBOHD-dependent ROS production and MPK3/6 activation also occur for extended periods during ETI [8], [9]. We previously reported that MAMPs can trigger accumulation of SA and thereby activate SA signaling [30], i.e., the EMT sectors positively regulate the SA sector. However, our network model contains negative links as well as positive ones between the sectors, suggesting that the regulatory relationships between the sectors can be positive or negative, depending on the context. In the following sections, we closely investigate this subnetwork of the EMT and SA sectors.

### The callose synthase PMR4 and the SA sector mutually inhibit each other

Callose deposition is a cell wall-based defense following recognition of pathogens [53]. PMR4 is the callose synthase responsible for callose deposition upon infection with pathogens or treatment with elicitors [52], [53], [54]. Our model predicted a negative relationship between PMR4 and SID2 (Figure 3A). It was previously reported that SA-mediated signaling is up-regulated in *pmr4-1* plants [52], i.e., PMR4 negatively regulates the SA sector, which can explain the predicted negative regulatory relationship between PMR4 and SID2. Can SA signaling also affect callose deposition? We quantified callose deposition in the SA sector mutants, *npr1-1* and *sid2-2*, after flg22 treatment according to the method described in Denoux *et al.* [55]. Together with the SA sector mutants, *pbs2-1* (a mutant with a RAR1 deletion) was included as a mutant with potentially enhanced callose deposition. RAR1 has a negative link with PMR4 in our network model, and different RAR1 alleles *rar1-20* and *rar1-29* were reported to have enhanced callose deposition phenotypes [56]. The callose deposition level in cotyledons of 10 day-old seedlings grown in liquid culture was measured at 6 and 16 hours post treatment (hpt) with 1µM flg22 (Figure 3B). Consistent with a previous report [45], no significant difference in the flg22-triggered callose deposition level was observed at 16 hpt between Col-0 wild type and the SA sector mutants. However, the callose deposition levels at 6 hpt in the SA sector mutants were significantly higher than in Col-0. At 6 hpt, the callose deposition level in Col-0 was not significantly different from the flg22-receptor mutant *fls2C*, so the Col-0 level was the background noise level. These results indicate that flg22-triggered callose deposition is enhanced in the SA sector mutants at an early time point: the SA sector negatively regulates the PMR4 sector. Thus, negative regulatory relationships between PMR4 and the SA sector are mutual.

There is also a positive relationship between PMR4 and NPR1. It has been reported that pretreatment with SA can compensate loss of the flg22-triggered callose deposition caused by a *pen2* mutation [45]. The positive PMR4-NPR1 link may correspond to this SA-enhanced callose deposition in *pen2* plants. Such context-dependent regulatory relationships involving PMR4 were anticipated as PMR4 has a higher number of links compared with other genes in our network model.

### The SA sector positively regulates RBOHD-dependent ROS production

ROS production minutes after treatment with flg22 is one of the very early MAMP-triggered responses. RBOHD is required for flg22-triggered ROS production [10]. A positive regulatory relationship between the SA sector and ROS production was predicted as RBOHD has a positive link with NPR1 in our network model (Figure 3A). We tested whether pretreatment with SA and/or a mutation in NPR1 affect flg22-triggered ROS production. Pretreatment of plant tissues with SA rather than co-treatment with SA and flg22 was chosen since flg22-triggered ROS production starts within a few minutes after addition of flg22. Col-0 and *npr1-1* were pretreated with 5µM SA or water for 3 hours before they were treated with 1µM flg22 or water. Pretreatment with SA enhanced ROS production in Col-0 wild type during the period between 3 and 12 minutes after treatment with flg22 (Table S7). This enhanced ROS production was abolished in *npr1-1*, which indicates that SA positively regulates ROS production in an NPR1-dependent manner. This observation is consistent with the model prediction of an NPR1-RBOHD positive regulatory relationship (Figure 2A).

### The EMT and the SA sectors negatively regulate each other in transcriptional activation of marker genes

**Figure 4:**
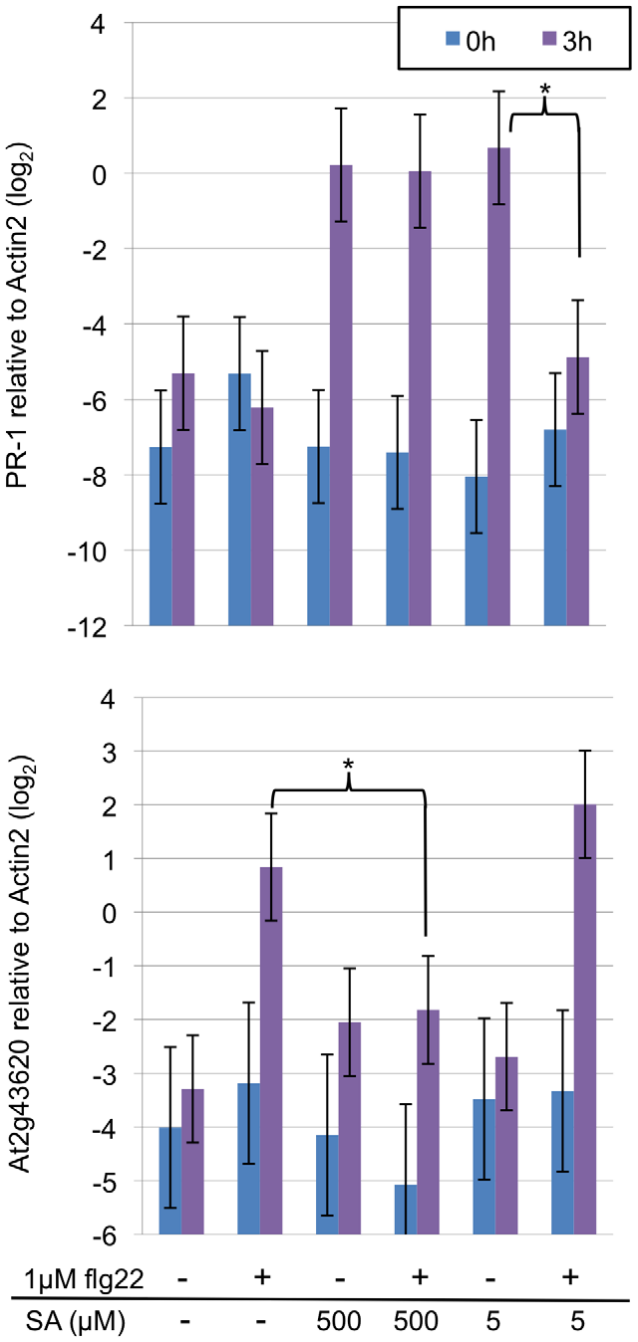
Mutual inhibition between the EMT and SA sectors. The mRNA levels of the *PR-1* and putative chitinase (At2g43620) genes were measured by qRT-PCR and used as proxies for the SA and EMT sector activities, respectively. The *Actin 2* mRNA level was used to normalize the mRNA measurements. Ten-day old seedlings were treated with indicated concentrations of SA and/or flg22 and harvested for mRNA measurements at the indicated times. *, *p*<0.05 for the indicated comparisons.

To further examine regulatory relationships between the EMT and the SA sectors, effects of SA and flg22 on the EMT and SA sectors, respectively, were examined using the mRNA level of a marker gene as a proxy for activity of each sector. Wild-type seedlings grown in liquid culture were treated with flg22 and/or SA. The mRNA levels of a putative chitinase (At3g43620) [30] and the *PR-1* (At2g14610) genes were quantified for the EMT and SA sector activities, respectively (Figure 4). Induction of SA accumulation by flg22 was not significant at 3 hpt [30]. We measured the marker gene mRNA levels up to 3 hpt, so SA accumulation caused by flg22 treatment was negligible. Treatment with 500 or 5 µM SA induced *PR-1* mRNA accumulation by 3 hpt. An inhibitory effect of 1 µM flg22 on *PR-1* mRNA induction was observed with 5 µM SA at 3 hpt but not with 500 µM SA. An inhibitory effect of 500 µM but not 5 µM SA on induction of the chitinase mRNA accumulation by 1 µM flg22 was observed at 3 hpt. Significant inhibitory effects of 1 µM flg22 and 500 or 5µM SA were not observed 1 or 2 hpt (Figure S4). Thus, the EMT and SA sectors have mutual inhibitory effects in a dose-dependent manner.

## Discussion

### Use of mRNA profiles as detailed descriptions of network states

We used mRNA profiles of mutant plants for inference of regulatory relationships among the genes corresponding to the mutations. This use of mRNA profiles was pioneered by the “compendium” approach [32], and further developed, for example, to the “connectivity map” approach [57]. However, these approaches focused on the most prominent similarities in the global space and did not intend to dissect combinations of similarities to reveal multi-dimensional similarity relationships among mRNA profiles. In our earlier work, we combined the LLE algorithm [33] and graphical representation to visualize differences among similarities in mRNA profiles of Arabidopsis mutants with variable local dimensionalities to reveal different mechanisms used in plant immunity [34]. However, the analysis in our earlier work was limited to the local space defined by the global distance. In the current study we used RepEdLEGG, in which LLE was recursively applied to the residual of the first round of LLE. This approach enabled us to detect weak regulatory relationships and to reveal a highly interconnected network structure.

A limitation of using mRNA profiles as descriptions of the network state is that the resolution of the network is determined by the number of network states measured – e.g., in our study, the number of Arabidopsis mutants profiled. It should be noted that in our network model, when the genes corresponding to the mutations were linked, the link means that the genes or some other network components near the genes in the actual signaling network have regulatory relationships.

On the other hand, an advantage of this approach is that the regulatory mode of the gene defined by a mutation does not have to be transcriptional although mRNA profiles are used for network inference. For example, we detected the regulatory relationship in the MPK6-EIN3 link even though MPK6 does not affect EIN3 expression, but rather its phosphorylation. Because the plant immune signaling network contains many major non-transcriptional regulatory components [9], [24], [43], [58], this advantage of the approach was essential for us to obtain a global network model using a single methodology.

Using the predicted relationships between the EMT and SA sectors as examples, we have demonstrated that the resulting undirected regulatory relationships are highly informative in generation of hypotheses to guide intensive studies in focused parts of the network. We built this highly informative model in a cost-effective manner: mRNA profiling using a small-scale array at a single time point under a single experimental condition. Therefore, applications of this approach should be beneficial in studies of complex signaling networks in any genetically tractable organisms.

Complex regulatory relationships among the network components strongly suggest that many relationships are dependent on context, such as the quantities and the states of other network components. To deepen our understanding of the signaling network, it will be important to elucidate the dynamic relationships among the network components. As the cost of mRNA profiling is rapidly decreasing, it will soon be practical to collect mRNA profiles of wild-type and many mutant plants at many time points. Such time-series mRNA profile data will enable extension of our network model to include information about network dynamics.

Furthermore, cost reduction in mRNA profiling will improve applications of the approach used in this study. First, it could allow a symmetric and highly-overlapping experiment group design, which would reduce potential biases in the data set. Second, it could allow inclusion of mRNA profiles from uninfected plants of all the genotypes. Inclusion of such profiles would enable separating the genotype effect and the genotype∶infection interaction for each profiled gene, which we cannot do with the current data set that only includes infected plants. However, expression level information from many genes is combined as the network state description in our approach. Different genes have different ratios between the genotype effect and the genotype∶infection interaction. A data set that includes information from such genes allows incorporation of information about the genotype effect and the genotype∶infection interaction in the network inference. This may have contributed to the success of our approach in the absence of mRNA profiles from uninfected plants. Third, cost reduction could allow profiling of many more genes. If many more genes are profiled, some aspects of the network states that evaded detection in mRNA profiles of a limited number of genes (571 genes in this study) may be detected, which could lead to discovery of additional weak regulatory relationships among the network components.

### Detection of weak regulatory relationships by RepEdLEGG

Implementation of RepEdLEGG was a key to building the highly interconnected network model. Thirty-two out of 67 links predicted were obtained in the second round of LLE using the residuals from the first round of LLE as the response. Eight out of the 32 links found in the second round were supported by previous evidence. These links found in the second round connect vertices whose global distances are not particularly small and represent weak regulatory relationships. The validities of many links found in the second round of LLE indicate that common multivariate analysis methods that depend solely on the global distance are not ideal for inference of a highly interconnected network.

Among existing methods, partial correlation is a method that can detect weak regulatory relationships [59], like RepEdLEGG. The partial correlation between vertices X and Y is defined, when all the other vertices are Z_1_, …, Z_n_, as the correlation between the residual of the linear regression of X with Z_1_, …, Z_n_ and the residual of the linear regression of Y with Z_1_, …, Z_n_. When the results of RepEdLEGG and the partial correlation were compared using the data set used in this study (*q*<0.01), 51 links were predicted in common (Figure S5). There were 16 and 5 links unique to RepEdLEGG and the partial correlation, respectively. Whereas 7 out of the 16 links uniquely predicted by RepEdLEGG had supporting literature evidence, none of the links unique to the partial correlation did. This result suggests a higher accuracy of inference by RepEdLEGG than by partial correlation. We speculate that the difference between the two methods resulted from a difference in the size of the space that is considered linear for each vertex. While RepEdLEGG constrains the linear space to that delimited by the neighboring vertices found in the first and second rounds of LLE, partial correlation assumes that the entire global space is linear. Although RepEdLEGG is hampered by the arbitrariness in determining the size of the linear space (i.e., determining the number of neighbor vertices), the superior performance of RepEdLEGG over the partial correlation suggests that constraining the size of the linear space is important in modeling of a complex regulatory network.

### Mutual inhibition between the EMT and SA sectors

Guided by our network model, we have demonstrated that the EMT and SA sectors can antagonize each other. Such mutual inhibition is not intuitive since both sectors positively contribute to resistance against *Pto* DC3000 [30], [45]. In addition, it appears to contradict our previous report that MAMPs trigger SA accumulation [30], which is equivalent to positive regulation of the SA sector by the EMT sectors. It should be noted that two important aspects, kinetic and quantitative effects, are overlooked in these simplified arguments. The induction of SA accumulation by flg22 clearly takes longer than 3 hpt [30] while the mutual inhibition between the EMT and the SA sectors was evident at 3 hpt (Figure 4). In addition, we observed dose dependence in the mutual inhibition: inhibition of the SA sector by flg22 was effective only when SA signaling was weak while inhibition of the EMT sectors by SA was effective only when SA signaling was strong (Figure 4). We think that such kinetic and quantitative effects play important roles in coordinating positive and negative regulatory relationships between these sectors.

**Figure 5:**
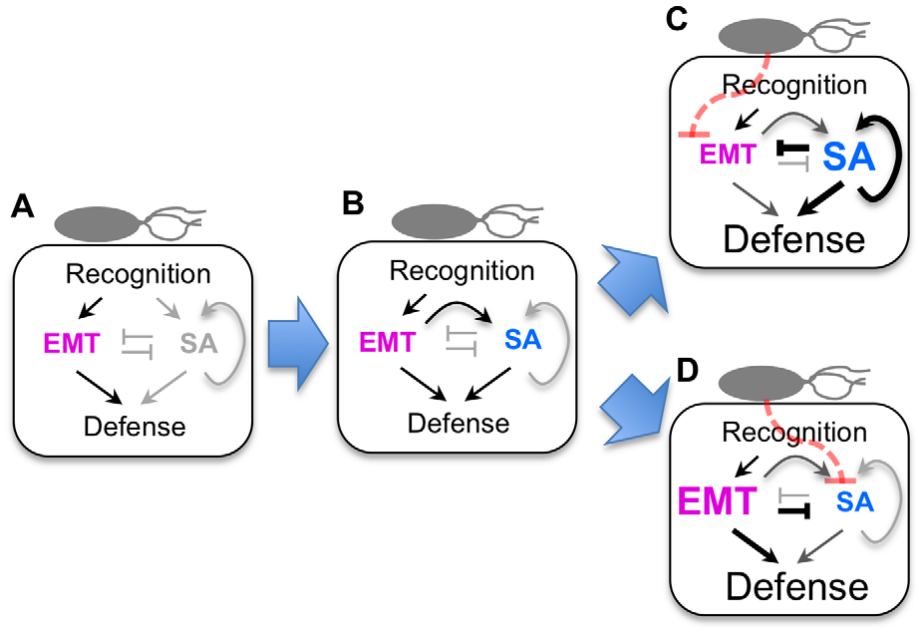
A hypothesis of sector switching. First, recognition of MAMPs leads to activation of the EMT sectors (A), which then activates the SA sector (B) [30]. If pathogen effectors perturb the EMT sectors, the inhibition of the SA sector by the EMT sectors becomes negligible, and the SA sector becomes highly activated, including signal amplification involving positive feedback [70], and deploys a potent defense response (C). If pathogen effectors perturb the SA sector, the inhibition of the EMT sectors by the SA sector becomes negligible, and the EMT sectors become highly activated and deploy a strong defense response (D). Note that the EMT sectors and the SA sector are not highly activated simultaneously.

The plant immune system must be robust against various perturbations caused by pathogens, which typically evolve much faster than plants. At the same time, not only are immune responses energy-expensive [60] but at least some are also detrimental to the plant fitness [61], [62], [63]. Therefore, ideally immune responses should be contained at the minimally necessary level. We speculate that to balance these apparently conflicting selection pressures, the EMT and SA sectors adjust the level of immune responses according to demand through the positive and negative regulatory relationships between them (Figure 5). When the plant is attacked by a pathogen, the EMT sectors are activated based on recognition of MAMPs. While the activation of the EMT sectors starts the activation of the SA sector with a delay, the SA sector does not become highly activated due to suppression by the strongly-activated EMT sectors. This is probably because detrimental effects of defense components controlled by the EMT sectors are less severe than those of the SA sector: if defense components controlled by the SA sector are not necessary, it is better not to activate them. The delay in activation of the SA sector by the EMT sectors is important in buying time for evaluation of the effect of the EMT sector-mediated defense. However, if the pathogen is to some extent adapted to the plant host and its effectors interfere with the EMT sectors, the resulting weakened activity of the EMT sectors could release the SA sector from suppression. In fact, several *P. syringae* effectors, such as HopAI1 [10], target components of the EMT sectors. Using the SA sector-controlled defense components against more virulent pathogens is reasonable, as the SA sector-controlled defenses are known to be potent in defense against biotrophic and hemi-biotrophic pathogens [39]. Thus, an elaborate combination of positive and negative regulatory relationships between the EMT and the SA sectors may enable shifting the balance between the EMT sectors for defense against less virulent pathogens to keep negative impacts of the immune response on plant fitness low and to reserve the SA sector for defense against more virulent biotrophic and hemi-biotrophic pathogens.

### The plant immune signaling network appears to have a sector-switching property

In our network model there are many inter-sector regulatory relationships. Such a high connectivity suggests a democratic network, in which each component of the network has a relatively small contribution to the function of the network and the level of contribution from each component is similar. We recently demonstrated that the AvrRpt2-ETI is robust against network perturbations because of positive contributions from each sector to immunity and compensatory interactions among them [31]. So, the network for AvrRpt2-ETI signaling appeared to be democratic. However, our current study showed that negative regulatory relationships are very common between different signaling sectors, such as between the EMT and the SA sectors. We speculate that the EMT and SA sectors are not exactly democratic: one of them is more active under a particular condition, and the other is suppressed by the active one; if the active sector is inhibited, the other sector gets activated to compensate. So, the apparent redundancy in immune signaling does not result from simple functional redundancy but from switching between the sectors. The prevalence of inter-sector negative regulatory relationships suggests that such sector-switching is common at the whole network level, not just between the EMT and SA sectors. In fact, an antagonistic relationship between the SA and JA sectors is well documented [64]. We propose to call this property of the signaling network “sector-switching”. If robustness of the immune system against fast-evolving pathogens had been the only driver in evolution, the signaling network could have evolved to be a simple redundant, democratic network. However, immune responses are generally deleterious to the host, and they impose fitness costs when the pressure from particular pathogens is not high [63]. Together with the demand to minimize negative impacts of immune response, we speculate that the signaling network has evolved to have a sector-switching property, so that the activities of the signaling sectors are switched in response to inputs to the network, such as inputs for induction of PTI and ETI, and to external perturbations, such as perturbations by pathogen effectors, to balance the performance and the negative impacts of the integrated immune system.

## Materials and Methods

### Plants and bacteria

All Arabidopsis plants, wild type and mutants, used in the study had the genetic background of accession Col-0. For mRNA profiling and ROS production assays, plants were grown in a controlled environment chamber at 22°C with 75% relative humidity and a 12h/12h light/dark cycle. For the assays using seedlings in liquid culture, seedlings were prepared essentially as described in Denoux *et al.* [55] with the following modifications: 0.25g/L as the concentration of sucrose in the culture medium, and the culture was incubated at 22°C. *Pseudomonas syringae* pv. *tomato* DC3000 carrying pLAFR3-*avrRpt2* (*Pto* DC3000 AvrRpt2) [65] was used for inoculation of plants subjected to mRNA profiling.

### Treatments

*Pto* DC3000 AvrRpt2 was cultured in King's B medium at room temperature (∼22°C) overnight and inocula were prepared at an OD_600_ of 0.05 in water. Leaves were infiltrated using a needle-less syringe as described in [66]. The flg22 peptide (QRLSTGSRINSAKDDAAGLQIA) was synthesized by EzBiolab Inc. (IN, USA) and was used at indicated concentrations. Sodium salicylate (Fisher Scientific, PA, USA) was used to prepare SA solutions at 5 or 500 µM. For treatment of seedlings, plates were centrifuged at 500 rpm for 10 seconds to remove condensation 1 day before treatment.

### mRNA profiling

Twenty-two mutants were divided into five experiment groups, and three biological replicates were made for each group, except for one (group 00) with two biological replicates. The data collection for the biological replicates was conducted at least one week apart. Each experiment group consisted of Col-0 in addition to seven mutants. Detailed information about grouping is provided in Table S1. The eight plants were grown at the outside positions of a 3×3 grid pattern in a 6″×6″ pot, and an additional Col-0 plant, which was not used for data collection, was grown in the center position of the grid pattern. The positions of the eight plants in each pot were randomly assigned. Some mutants in these experiments were irrelevant to this study and were excluded from analyses following normalization of mRNA profiles. The 5^th^ experiment group (group 00) consisting of one or two mutants used in each of three experiment groups (groups 01, 02, and 03) and Col-0 was included to reduce potential bias associated with the experiment groups, e.g., biases associated with particular dates when experiments were conducted or particular combinations of genotypes tested together.

Two fully-developed leaves of each 4 week-old plant were inoculated with *Pto* DC3000 AvrRpt2. For each mRNA profile, inoculated leaves were harvested from three plants of the same genotype from three different pots at 6 hpi and pooled.

Procedures from target preparation to microarray data collection were performed as described in Sato *et al.* [37].

### Data preprocessing

Raw expression data were normalized using the stable gene-based quantile normalization (SBQ) method [37]. For comparison of profiles among different plant genotypes tested in different experiment groups, it was necessary to compensate for potential bias caused by separating genotypes to different groups. A 2-stage mixed effect linear model was fitted to the data from each experiment group separately:
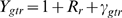


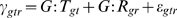
where *Y*, *G*, *T*, *R*, *γ*, and *ε* are log_2_-transformed expression level value, gene, genotype, replicate, residual of the 1^st^ model, and residual of the 2^nd^ model. *G* and *T* are fixed effects, and *R*, *γ*, and *ε* are random effects. The second model was fitted for each gene separately.

Using the *G*:*T* values for the genotypes common between pairs of the experiment groups, calibration values among the experiment groups were calculated for each gene. The values in the initial SBQ-normalized data set containing all the experiment groups were corrected using the calibration values and were used to fit another 2-stage model:
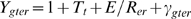


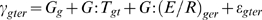
where *Y*, *G*, *T*, *E*, *R*, *γ*, and *ε* are log_2_-transformed expression level value, gene, genotype, experiment group, replicate, residual of the 1^st^ model, and residual of the 2^nd^ model. *G* and *T* are fixed effects, and *E*, *R*, *γ*, and *ε* are random effects. The second model was fitted for each gene separately. The contrasts in the model were made to obtain the difference value between each mutant and Col-0 in each *T_t_* + *G*:*T_gt_*.

### Network inference by RepEdLEGG

A data set with 480 genes each of which had at least one mutant genotype with the significant log_2_-transformed ratio value (*q*<0.05) were used to compare mRNA profiles of the genotypes (480 genes × 22 genotypes). The log_2_-transformed ratio values were not centered but scaled across the genes for each genotype (difference profiles). In this way, the order of the pairwise distances of the genotype difference profiles is invariant when either the uncentered Pearson correlation coefficient or the Euclidean distance is used. EdLEGG was modified from LEGG [36] to use the Euclidean distance instead of the uncentered Pearson correlation, so that multiple regression can be used for the calculation. Briefly, in a data set of *n* genes × *m* genotypes, the difference profile of genotype *i* is denoted as a vector 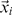 in an *n*-dimensional space. For the vector of each genotype *i*, *k* closest neighboring genotype vectors 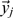 were identified using the uncentered Pearson correlation coefficient. *P_i_* is the set of such *j* (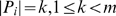). The value *k* defines the size of the local space. Then the following multiple regression was fitted by minimizing the residual vector size 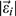:
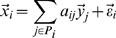
In this first round of EdLEGG, the condition, 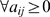, was applied to allow only positive regulatory relationships for the identification of major components illustrated in Figure S1B. *k* = 6 was used in this study as this made some of *a_ij_* for most *i* insignificant, which suggests that each local space was sufficiently sampled.

In RepEdLEGG, each residual vector 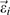 was subjected to a second round of EdLEGG. For each 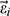, *l* closest neighboring genotype vectors 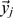 were identified using the absolute value of the uncentered Pearson correlation coefficient. *Q_i_* is the set of such *j* (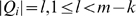). In this way, the genotype vectors that are negatively correlated as well as positively correlated can be identified as neighbors, which allows detection of both negative and positive regulatory relationships. The following multiple regression was fitted by minimizing the residual vector size 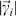:
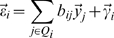
In this second round, the coefficients *b_ij_* were allowed to take positive or negative values to include negative regulatory relationships. *l* = 5 was used in this study as this made some of *b_ij_* for most *i* insignificant, which suggests that each local space was sufficiently sampled. The *p*-value associated with each of the coefficients *a_ij_* and *b_ij_*, obtained from multiple regression, was corrected using the Benjamini-Hochberg False Discovery Rate (FDR) [67] to obtain the *q*-value, and the neighboring genotype vectors with coefficients significant for the indicated *q*-value threshold, 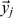, were identified for each genotype *i* (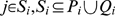).

The output of RepEdLEGG was further evaluated using a leave-one-out (LOO) cross-validation. In each case, the profile for one of the 22 mutants was removed from the data set, and this LOO data set was subjected to RepEdLEGG analysis. Links that were found in at least 18 LOO cross-validation cases were considered significant. Note that for a particular link, 20 LOO data sets have both the genotypes flanking the link.

Then, all the LOO-filtered neighboring genotype vectors from both rounds were subjected to multiple regression together to obtain the final coefficients *c_ij_*, which could be positive or negative, and their associated *p*-values by minimizing the residual vector size, 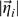:
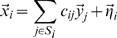
The obtained *p*-value was FDR-corrected to obtain the *q*-value. When two significant coefficients were found for a single link (*a_ij_* and *a_ji_*), the coefficient with the smaller *q*-value was selected.

In the model, the significant links between the mutant genotypes are represented as the links between the genes corresponding to the mutations. The links are color-coded in Figure 2 according to their associated coefficient values.

### Literature analysis

To collect experimentally validated regulatory relationships, a systematic search of literature describing the 22 genes in our network model was conducted. “LocusPublished.20091204.txt” in TAIR (ftp://ftp.arabidopsis.org/home/tair/User_Requests/LocusPublished.20091204.txt) was used to map genes to literature. A custom Perl script was used to parse information about each gene of interest in the file to identify publications, each of which was simultaneously mapped to any pair of the 22 genes, and to generate hyperlinks to the PubMed records (http://www.ncbi.nlm.nih.gov/pubmed/) for the identified publications. Next, the contents of the identified publications were inspected for appropriateness. This relatively unbiased procedure identified 22 known regulatory relationships. Three more known regulatory relationships were added based on publications that were not included in “LocusPublished.20091204.txt” but that we knew. To our knowledge, these 25 relationships are the only relationships known for the 22 genes.

### Callose deposition assay

Ten day-old Col-0 seedlings grown in liquid culture were incubated with 1 µM flg22 for 6 or 16 hours. Cotyledons were harvested for staining with aniline blue. Staining and visualization procedures were described in Wang *et al.* [68]. One image was obtained from each cotyledon. Stained callose deposits were counted using a custom macro combined with a custom plug-in for Image J (http:// rsb.info.nih.gov/ij/). The macro performs noise reduction, binarizing images, and counting objects with filtering for a particular size range.

### ROS production assay

Six week-old adult plants grown under the conditions described above were used. Eight leaf discs with a diameter of 4 mm were prepared and incubated for ∼15 hours in sterilized water in 24-well flat-bottom cell culture plates (Corning, Inc., MA, USA) before pre-treatments with water or 5 µM SA. Leaf discs for mock and SA pre-treatments were collected from each half of the same leaves. Eight leaf discs were used for a single sample, and four replicated samples were made using different individual plants for each combination of genotype and treatment. Leaf disks pre-treated for 3 hours were then treated with 1 µM flg22 or water. These were considered to be four conditions: 2 pre-treatments×2 treatments. The ROS production level was measured as the relative luminescence value as described in Trujiro *et al.* [69]. The results were analyzed by fitting a polynomial linear model through the ROS production curves of individual measurements and using a mixed-effect linear model on the coefficients of these curves [36]:
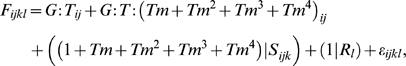
where *F*, *G*, *T*, *Tm*, *S*, *R*, and *ε* are measured ROS production value, genotype, condition, time, sample, replicate, and residual, respectively. *G*, *T*, and *Tm* are fixed effects, and *S*, *R*, and *ε* are random effects. To avoid convergence problems, the coefficients of the (1+*Tm*+*Tm* ^2^+*Tm* ^3^+*Tm* ^4^)|*S_ijk_* random effect were assumed to be independent and time was centered and scaled to range from −1 to 1.

### flg22-SA competition assay

Ten-day old Col-0 seedlings were treated with SA at an indicated concentration and/or 1 µM flg22, or water for 3 hours and harvested for RNA extraction. RNA extraction and quantitative RT-PCR were performed as described in Tsuda *et al.* [31]. The C_t_ values of a putative chitinase (At3g43620) and PR-1 relative to Actin2 (At2g18780) were fitted to a mixed linear model:
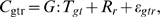
where *C*, *G*:*T*, *R*, and *ε* are relative C_t_ value, gene∶treatment interaction, replicate effect and residual, respectively. *G* and *T* are fixed effects, and *R* and *ε* are random effects. The mean estimate of the gene∶treatment interaction was used as the modeled C_t_ value. For the *t*-tests, the standard error appropriate for each comparison was calculated using the variance and covariance values obtained from the model fitting.

### Accession numbers

The Gene Expression Omnibus (GEO) (http://www.ncbi.nlm.nih.gov/geo) accession numbers for data discussed in this paper are GSE19663 and GSM490922 to GSM490978.

## Supporting Information

Figure S1 Analysis of residuals from the first round of LLE in RepEdLEGG allows detection of minor similarity (Conceptual diagram). For the sake of visualization, log-transformed expression level ratio values of two genes (i.e., two dimensions) are plotted for 70 mutant plants, which correspond to the data points. (A) Major similarities among the data points can be identified as clusters in the global space. Four clusters are indicated by different colors of the data points. (B) For each of the red and blue clusters, major components (thick gray arrows) are identified. This can be achieved by the first round of LLE. (C) Once the major components are subtracted from the red and blue clusters (i.e., residuals), minor similarities (the star-like shapes of the clusters) can be identified. Note that since it is impossible to represent events in a high-dimensional space accurately in a two-dimensional space, this figure is by no means an accurate representation of the RepEdLEGG procedure. Instead, the purpose of this figure is to illustrate the idea that analysis of residuals allows detection of minor similarities in expression profile data, which correspond to weak regulatory relationships among the genes corresponding to the mutations.
(4.81 MB TIF)

Figure S2 Categories of links inferred by RepEdLEGG. (A) Links inferred by the RepEdLEGG procedure are classified into those inferred in the first and second rounds of the LLE procedure. The links inferred in the second round are further divided into positive and negative links. (B) Links with supporting, conflicting or no evidence. The chart in the inset indicates the proportion of evidence-supported links inferred in the first and second rounds. 1^st^ evidence (+), evidence-supported links inferred in the first round of LLE; 2^nd^+ evidence (+), evidence-supported links for positive regulatory relationships inferred in the second round of LLE; 2^nd^- evidence (+), evidence-supported links for negative regulatory relationships inferred in the second round of LLE. Supporting evidence is listed in Table S3.
(0.24 MB TIF)

Figure S3 Analyses of the link specificities. (A and A′) The link between EIN3 and MPK6 is not ET signaling-dependent. To test specificity of the link between EIN3 and MPK6, the *ein3* profile was removed from the data set, and the RepEdLEGG analysis was performed. A, The links involving EIN3 are highlighted in the full model (Figure 2C). A′, The links involving EIN2 are highlighted in the model with EIN3 removed. (B and B′) The NO sector has positive relationships with the SA sector. To analyze the specificity of links between the NO sector components (NOA1 and NIA2) and the SA sector components, the RepEdLEGG analysis was performed with the data set with the *noa1* profile removed. B, The links involving the NO signaling components are highlighted in the full model (Figure 2C). B′, The links involving NIA2 are highlighted in the model without NOA1. Links inferred 17 times in the LOO cross-validation results were considered significant when a data set with one mutant profile removed is used. Note that a link can be inferred 19 times at maximum when one component is removed. The color codes of the links were determined based on coefficients associated with the links. The color codes for the vertices at the bottom of the figure show the signaling sector assignments of the genes corresponding to the mutations.
(1.01 MB TIF)

Figure S4 Time-course analysis of mutual inhibition between the EMT and SA sectors. The mRNA levels of the *PR-1* and putative chitinase (At2g43620) genes were measured by qRT-PCR and used as proxies of the SA and EMT sector activities, respectively. The *Actin 2* mRNA level was used to normalize the mRNA measurements. Ten-day old seedlings were treated with indicated concentrations of SA and/or flg22 and harvested for mRNA measurements at the indicated times. *, *p*<0.05 for the indicated comparisons.
(0.32 MB TIF)

Figure S5 Comparison of RepEdlEGG with partial correlation. The expression ratios between mutants and Col-0 wild-type (22 genotypes×480 genes) were analyzed using RepEdLEGG and partial correlation with LOO cross-validation. Links inferred 18 times in the LOO results were considered significant.
(0.19 MB TIF)

Table S1 Experiment groups in mRNA profiling
(0.03 MB XLS)

Table S2 Statistics for the Arabidopsis immune signaling network model
(0.04 MB XLS)

Table S3 Regulatory relationships among the 22 genes supported by literature
(0.03 MB XLS)

Table S4 The EIN3-SID2 relationship is masked by JAR1 in the full model.
(0.04 MB XLS)

Table S5 Statistics for the network model without EIN3 (for Figs. S3 A and A′)
(0.04 MB XLS)

Table S6 Statistics for the network model without NOA1 (for Figs. S3 B and B′)
(0.04 MB XLS)

Table S7 Statistics for effects of SA pretreatment on flg22-triggered ROS production
(0.03 MB XLS)

## References

[1] Bar-Yam Y, Harmon D, de Bivort B (2009). Systems biology. Attractors and democratic dynamics.. Science.

[2] Kitano H (2002). Systems biology: a brief overview.. Science.

[3] Jones JD, Dangl JL (2006). The plant immune system.. Nature.

[4] Gómez-Gómez L, Boller T (2000). FLS2: an LRR receptor-like kinase involved in the perception of the bacterial elicitor flagellin in Arabidopsis.. Mol Cell.

[5] Block A, Li G, Fu ZQ, Alfano JR (2008). Phytopathogen type III effector weaponry and their plant targets.. Curr Opin Plant Biol.

[6] Axtell MJ, Staskawicz BJ (2003). Initiation of RPS2-specified disease resistance in Arabidopsis is coupled to the AvrRpt2-directed elimination of RIN4.. Cell.

[7] Mackey D, Belkhadir Y, Alonso JM, Ecker JR, Dangl JL (2003). Arabidopsis RIN4 is a target of the type III virulence effector AvrRpt2 and modulates RPS2-mediated resistance.. Cell.

[8] Underwood W, Zhang S, He SY (2007). The Pseudomonas syringae type III effector tyrosine phosphatase HopAO1 suppresses innate immunity in Arabidopsis thaliana.. Plant J.

[9] Torres MA, Dangl JL, Jones JD (2002). Arabidopsis gp91phox homologues AtrbohD and AtrbohF are required for accumulation of reactive oxygen intermediates in the plant defense response.. Proc Natl Acad Sci USA.

[10] Zhang J, Shao F, Li Y, Cui H, Chen L (2007). A Pseudomonas syringae effector inactivates MAPKs to suppress PAMP-induced immunity in plants.. Cell Host Microbe.

[11] Zeier J, Delledonne M, Mishina T, Severi E, Sonoda M (2004). Genetic elucidation of nitric oxide signaling in incompatible plant-pathogen interactions.. Plant Physiol.

[12] Zeidler D, Zähringer U, Gerber I, Dubery I, Hartung T (2004). Innate immunity in Arabidopsis thaliana: lipopolysaccharides activate nitric oxide synthase (NOS) and induce defense genes.. Proc Natl Acad Sci USA.

[13] Navarro L, Zipfel C, Rowland O, Keller I, Robatzek S (2004). The transcriptional innate immune response to flg22. Interplay and overlap with Avr gene-dependent defense responses and bacterial pathogenesis.. Plant Physiol.

[14] Loake G, Grant M (2007). Salicylic acid in plant defence–the players and protagonists.. Curr Opin Plant Biol.

[15] Pozo MJ, Van Loon LC, Pieterse CM (2004). Jasmonates - Signals in Plant-Microbe Interactions.. Journal of Plant Growth and Regulation.

[16] van Loon LC, Geraats BP, Linthorst HJ (2006). Ethylene as a modulator of disease resistance in plants.. Trends Plant Sci.

[17] Wildermuth MC, Dewdney J, Wu G, Ausubel FM (2001). Isochorismate synthase is required to synthesize salicylic acid for plant defence.. Nature.

[18] Nawrath C, Heck S, Parinthawong N, Métraux JP (2002). EDS5, an essential component of salicylic acid-dependent signaling for disease resistance in Arabidopsis, is a member of the MATE transporter family.. Plant Cell.

[19] Cao H, Glazebrook J, Clarke JD, Volko S, Dong X (1997). The Arabidopsis NPR1 gene that controls systemic acquired resistance encodes a novel protein containing ankyrin repeats.. Cell.

[20] Falk A, Feys BJ, Frost LN, Jones JD, Daniels MJ (1999). EDS1, an essential component of R gene-mediated disease resistance in Arabidopsis has homology to eukaryotic lipases.. Proc Natl Acad Sci USA.

[21] Jirage D, Tootle TL, Reuber TL, Frost LN, Feys BJ (1999). Arabidopsis thaliana PAD4 encodes a lipase-like gene that is important for salicylic acid signaling.. Proc Natl Acad Sci USA.

[22] Glazebrook J, Chen W, Estes B, Chang HS, Nawrath C (2003). Topology of the network integrating salicylate and jasmonate signal transduction derived from global expression phenotyping.. Plant J.

[23] Staswick PE, Tiryaki I, Rowe ML (2002). Jasmonate response locus JAR1 and several related Arabidopsis genes encode enzymes of the firefly luciferase superfamily that show activity on jasmonic, salicylic, and indole-3-acetic acids in an assay for adenylation.. Plant Cell.

[24] Thines B, Katsir L, Melotto M, Niu Y, Mandaokar A (2007). JAZ repressor proteins are targets of the SCF(COI1) complex during jasmonate signalling.. Nature.

[25] Lorenzo O, Chico JM, Sánchez-Serrano JJ, Solano R (2004). JASMONATE-INSENSITIVE1 encodes a MYC transcription factor essential to discriminate between different jasmonate-regulated defense responses in Arabidopsis.. Plant Cell.

[26] Alonso JM, Hirayama T, Roman G, Nourizadeh S, Ecker JR (1999). EIN2, a bifunctional transducer of ethylene and stress responses in Arabidopsis.. Science.

[27] Chao Q, Rothenberg M, Solano R, Roman G, Terzaghi W (1997). Activation of the ethylene gas response pathway in Arabidopsis by the nuclear protein ETHYLENE-INSENSITIVE3 and related proteins.. Cell.

[28] Robert-Seilaniantz A, Navarro L, Bari R, Jones JD (2007). Pathological hormone imbalances.. Curr Opin Plant Biol.

[29] Zipfel C, Robatzek S, Navarro L, Oakeley EJ, Jones JD (2004). Bacterial disease resistance in Arabidopsis through flagellin perception.. Nature.

[30] Tsuda K, Sato M, Glazebrook J, Cohen JD, Katagiri F (2008). Interplay between MAMP-triggered and SA-mediated defense responses.. Plant J.

[31] Tsuda K, Sato M, Stoddard T, Glazebrook J, Katagiri F (2009). Network properties of robust immunity in plants.. PLoS Genet.

[32] Hughes TR, Marton MJ, Jones AR, Roberts CJ, Stoughton R (2000). Functional discovery via a compendium of expression profiles.. Cell.

[33] Roweis ST, Saul LK (2000). Nonlinear dimensionality reduction by locally linear embedding.. Science.

[34] Katagiri F, Glazebrook J (2003). Local Context Finder (LCF) reveals multidimensional relationships among mRNA expression profiles of Arabidopsis responding to pathogen infection.. Proc Natl Acad Sci USA.

[35] van Leeuwen H, Kliebenstein DJ, West MA, Kim K, van Poecke R (2007). Natural variation among Arabidopsis thaliana accessions for transcriptome response to exogenous salicylic acid.. Plant Cell.

[36] Van Poecke RM, Sato M, Lenarz-Wyatt L, Weisberg S, Katagiri F (2007). Natural variation in RPS2-mediated resistance among Arabidopsis accessions: correlation between gene expression profiles and phenotypic responses.. Plant Cell.

[37] Sato M, Mitra RM, Coller J, Wang D, Spivey NW (2007). A high-performance, small-scale microarray for expression profiling of many samples in Arabidopsis-pathogen studies.. Plant J.

[38] Delledonne M, Xia Y, Dixon RA, Lamb C (1998). Nitric oxide functions as a signal in plant disease resistance.. Nature.

[39] Glazebrook J (2005). Contrasting mechanisms of defense against biotrophic and necrotrophic pathogens.. Annu Rev Phytopathol.

[40] Dangl JL, Jones JD (2001). Plant pathogens and integrated defence responses to infection.. Nature.

[41] He P, Chintamanani S, Chen Z, Zhu L, Kunkel BN (2004). Activation of a COI1-dependent pathway in Arabidopsis by Pseudomonas syringae type III effectors and coronatine.. Plant J.

[42] Gómez-Gómez L, Felix G, Boller T (1999). A single locus determines sensitivity to bacterial flagellin in Arabidopsis thaliana.. Plant J.

[43] Asai T, Tena G, Plotnikova J, Willmann MR, Chiu WL (2002). MAP kinase signalling cascade in Arabidopsis innate immunity.. Nature.

[44] Bauer Z, Gómez-Gómez L, Boller T, Felix G (2001). Sensitivity of different ecotypes and mutants of Arabidopsis thaliana toward the bacterial elicitor flagellin correlates with the presence of receptor-binding sites.. J Biol Chem.

[45] Clay NK, Adio AM, Denoux C, Jander G, Ausubel FM (2009). Glucosinolate metabolites required for an Arabidopsis innate immune response.. Science.

[46] Tao Y, Xie Z, Chen W, Glazebrook J, Chang HS (2003). Quantitative nature of Arabidopsis responses during compatible and incompatible interactions with the bacterial pathogen Pseudomonas syringae.. Plant Cell.

[47] Takahashi F, Yoshida R, Ichimura K, Mizoguchi T, Seo S (2007). The mitogen-activated protein kinase cascade MKK3-MPK6 is an important part of the jasmonate signal transduction pathway in Arabidopsis.. Plant Cell.

[48] Chen H, Xue L, Chintamanani S, Germain H, Lin H (2009). ETHYLENE INSENSITIVE3 and ETHYLENE INSENSITIVE3-LIKE1 repress SALICYLIC ACID INDUCTION DEFICIENT2 expression to negatively regulate plant innate immunity in Arabidopsis.. Plant Cell.

[49] Yoo SD, Cho YH, Tena G, Xiong Y, Sheen J (2008). Dual control of nuclear EIN3 by bifurcate MAPK cascades in C2H4 signalling.. Nature.

[50] Zottini M, Costa A, De Michele R, Ruzzene M, Carimi F (2007). Salicylic acid activates nitric oxide synthesis in Arabidopsis.. J Exp Bot.

[51] Nawrath C, Métraux JP (1999). Salicylic acid induction-deficient mutants of Arabidopsis express PR-2 and PR-5 and accumulate high levels of camalexin after pathogen inoculation.. Plant Cell.

[52] Nishimura MT, Stein M, Hou BH, Vogel JP, Edwards H (2003). Loss of a callose synthase results in salicylic acid-dependent disease resistance.. Science.

[53] Ham JH, Kim MG, Lee SY, Mackey D (2007). Layered basal defenses underlie non-host resistance of Arabidopsis to Pseudomonas syringae pv. phaseolicola.. Plant J.

[54] Jacobs AK, Lipka V, Burton RA, Panstruga R, Strizhov N (2003). An Arabidopsis Callose Synthase, GSL5, Is Required for Wound and Papillary Callose Formation.. Plant Cell.

[55] Denoux C, Galletti R, Mammarella N, Gopalan S, Werck D (2008). Activation of Defense Response Pathways by OGs and Flg22 Elicitors in Arabidopsis Seedlings.. Mol Plant.

[56] Shang Y, Li X, Cui H, He P, Thilmony R (2006). RAR1, a central player in plant immunity, is targeted by Pseudomonas syringae effector AvrB.. Proc Natl Acad Sci USA.

[57] Lamb J, Crawford ED, Peck D, Modell JW, Blat IC (2006). The Connectivity Map: using gene-expression signatures to connect small molecules, genes, and disease.. Science.

[58] Dong X (2004). NPR1, all things considered.. Curr Opin Plant Biol.

[59] Toh H, Horimoto K (2002). Inference of a genetic network by a combined approach of cluster analysis and graphical Gaussian modeling.. Bioinformatics.

[60] Bolton MD (2009). Primary metabolism and plant defense–fuel for the fire.. Mol Plant Microbe Interact.

[61] Bowling SA, Clarke JD, Liu Y, Klessig DF, Dong X (1997). The cpr5 mutant of Arabidopsis expresses both NPR1-dependent and NPR1-independent resistance.. Plant Cell.

[62] Clarke JD, Liu Y, Klessig DF, Dong X (1998). Uncoupling PR gene expression from NPR1 and bacterial resistance: characterization of the dominant Arabidopsis cpr6-1 mutant.. Plant Cell.

[63] Tian D, Traw MB, Chen JQ, Kreitman M, Bergelson J (2003). Fitness costs of R-gene-mediated resistance in Arabidopsis thaliana.. Nature.

[64] Spoel SH, Koornneef A, Claessens SM, Korzelius JP, Van Pelt JA (2003). NPR1 modulates cross-talk between salicylate- and jasmonate-dependent defense pathways through a novel function in the cytosol.. Plant Cell.

[65] Whalen MC, Innes RW, Bent AF, Staskawicz BJ (1991). Identification of Pseudomonas syringae pathogens of Arabidopsis and a bacterial locus determining avirulence on both Arabidopsis and soybean.. Plant Cell.

[66] Katagiri F, Thilmony R, He SY (2002). The Arabidopsis Thaliana-Pseudomonas Syringae Interaction.

[67] Benjamini Y, Hochberg Y (1995). Controlling the False Discovery Rate: a Practical and Powerful Approach to Multiple Testing.. Journal of the Royal Statistical Society B.

[68] Wang L, Tsuda K, Sato M, Cohen JD, Katagiri F (2009). Arabidopsis CaM binding protein CBP60g contributes to MAMP-induced SA accumulation and is involved in disease resistance against Pseudomonas syringae.. PLoS Pathog.

[69] Trujillo M, Ichimura K, Casais C, Shirasu K (2008). Negative regulation of PAMP-triggered immunity by an E3 ubiquitin ligase triplet in Arabidopsis.. Curr Biol.

[70] Shah J (2003). The salicylic acid loop in plant defense.. Curr Opin Plant Biol.

[71] Guo FQ, Okamoto M, Crawford NM (2003). Identification of a plant nitric oxide synthase gene involved in hormonal signaling.. Science.

[72] Torres MA, Onouchi H, Hamada S, Machida C, Hammond-Kosack KE (1998). Six Arabidopsis thaliana homologues of the human respiratory burst oxidase (gp91phox).. Plant J.

[73] Xie DX, Feys BF, James S, Nieto-Rostro M, Turner JG (1998). COI1: an Arabidopsis gene required for jasmonate-regulated defense and fertility.. Science.

[74] Park JH, Halitschke R, Kim HB, Baldwin IT, Feldmann KA (2002). A knock-out mutation in allene oxide synthase results in male sterility and defective wound signal transduction in Arabidopsis due to a block in jasmonic acid biosynthesis.. Plant J.

[75] Wang H, Ngwenyama N, Liu Y, Walker JC, Zhang S (2007). Stomatal development and patterning are regulated by environmentally responsive mitogen-activated protein kinases in Arabidopsis.. Plant Cell.

[76] Liu Y, Zhang S (2004). Phosphorylation of 1-aminocyclopropane-1-carboxylic acid synthase by MPK6, a stress-responsive mitogen-activated protein kinase, induces ethylene biosynthesis in Arabidopsis.. Plant Cell.

[77] Kang L, Li J, Zhao T, Xiao F, Tang X (2003). Interplay of the Arabidopsis nonhost resistance gene NHO1 with bacterial virulence.. Proc Natl Acad Sci U S A.

[78] Wilkinson JQ, Crawford NM (1991). Identification of the Arabidopsis CHL3 gene as the nitrate reductase structural gene NIA2.. Plant Cell.

[79] Century KS, Shapiro AD, Repetti PP, Dahlbeck D, Holub E (1997). NDR1, a pathogen-induced component required for Arabidopsis disease resistance.. Science.

[80] Warren RF, Merritt PM, Holub E, Innes RW (1999). Identification of three putative signal transduction genes involved in R gene-specified disease resistance in Arabidopsis.. Genetics.

[81] Tornero P, Merritt P, Sadanandom A, Shirasu K, Innes RW (2002). RAR1 and NDR1 contribute quantitatively to disease resistance in Arabidopsis, and their relative contributions are dependent on the R gene assayed.. Plant Cell.

[82] Lipka V, Dittgen J, Bednarek P, Bhat R, Wiermer M (2005). Pre- and postinvasion defenses both contribute to nonhost resistance in Arabidopsis.. Science.

[83] Bent AF, Kunkel BN, Dahlbeck D, Brown KL, Schmidt R (1994). RPS2 of Arabidopsis thaliana: a leucine-rich repeat class of plant disease resistance genes.. Science.

[84] Mindrinos M, Katagiri F, Yu GL, Ausubel FM (1994). The A. thaliana disease resistance gene RPS2 encodes a protein containing a nucleotide-binding site and leucine-rich repeats.. Cell.

[85] Feys BJ, Wiermer M, Bhat RA, Moisan LJ, Medina-Escobar N (2005). Arabidopsis SENESCENCE-ASSOCIATED GENE101 stabilizes and signals within an ENHANCED DISEASE SUSCEPTIBILITY1 complex in plant innate immunity.. Plant Cell.

[86] Lozano-Juste J, León J (2010). Enhanced abscisic acid-mediated responses in nia1nia2noa1-2 triple mutant impaired in NIA/NR- and AtNOA1-dependent nitric oxide biosynthesis in Arabidopsis.. Plant Physiol.

[87] Gfeller A, Liechti R, Farmer EE (2010). Arabidopsis jasmonate signaling pathway.. Science signaling.

[88] Laudert D, Weiler EW (1998). Allene oxide synthase: a major control point in Arabidopsis thaliana octadecanoid signalling.. Plant J.

[89] Roman G, Lubarsky B, Kieber JJ, Rothenberg M, Ecker JR (1995). Genetic analysis of ethylene signal transduction in Arabidopsis thaliana: five novel mutant loci integrated into a stress response pathway.. Genetics.

[90] Chini A, Fonseca S, Fernández G, Adie B, Chico JM (2007). The JAZ family of repressors is the missing link in jasmonate signalling.. Nature.

[91] Yan Y, Stolz S, Chételat A, Reymond P, Pagni M (2007). A downstream mediator in the growth repression limb of the jasmonate pathway.. Plant Cell.

[92] Laurie-Berry N, Joardar V, Street IH, Kunkel BN (2006). The Arabidopsis thaliana JASMONATE INSENSITIVE 1 gene is required for suppression of salicylic acid-dependent defenses during infection by Pseudomonas syringae.. Mol Plant Microbe Interact.

[93] Bright J, Desikan R, Hancock JT, Weir IS, Neill SJ (2006). ABA-induced NO generation and stomatal closure in Arabidopsis are dependent on H2O2 synthesis.. Plant J.

[94] Cao H, Bowling SA, Gordon AS, Dong X (1994). Characterization of an Arabidopsis Mutant That Is Nonresponsive to Inducers of Systemic Acquired Resistance.. Plant Cell.

[95] Zhou N, Tootle TL, Tsui F, Klessig DF, Glazebrook J (1998). PAD4 functions upstream from salicylic acid to control defense responses in Arabidopsis.. Plant Cell.

[96] Belkhadir Y, Nimchuk Z, Hubert DA, Mackey D, Dangl JL (2004). Arabidopsis RIN4 negatively regulates disease resistance mediated by RPS2 and RPM1 downstream or independent of the NDR1 signal modulator and is not required for the virulence functions of bacterial type III effectors AvrRpt2 or AvrRpm1.. Plant Cell.

[97] Wang L, Mitra RM, Hasselmann KD, Sato M, Lenarz-Wyatt L (2008). The Genetic Network Controlling the Arabidopsis Transcriptional Response to Pseudomonas syringae pv. maculicola: Roles of Major Regulators and the Phytotoxin Coronatine.. Mol Plant Microbe Interact.

