# Supplementary figures and images for: Network Modeling Reveals Prevalent Negative Regulatory Relationships between Signaling Sectors in Arabidopsis Immune Signaling
Source: PLoS Pathog. 2010 Jul 22;6(7):e1001011. doi: 10.1371/journal.ppat.1001011 (PMC2908620; doi:10.1371/journal.ppat.1001011)

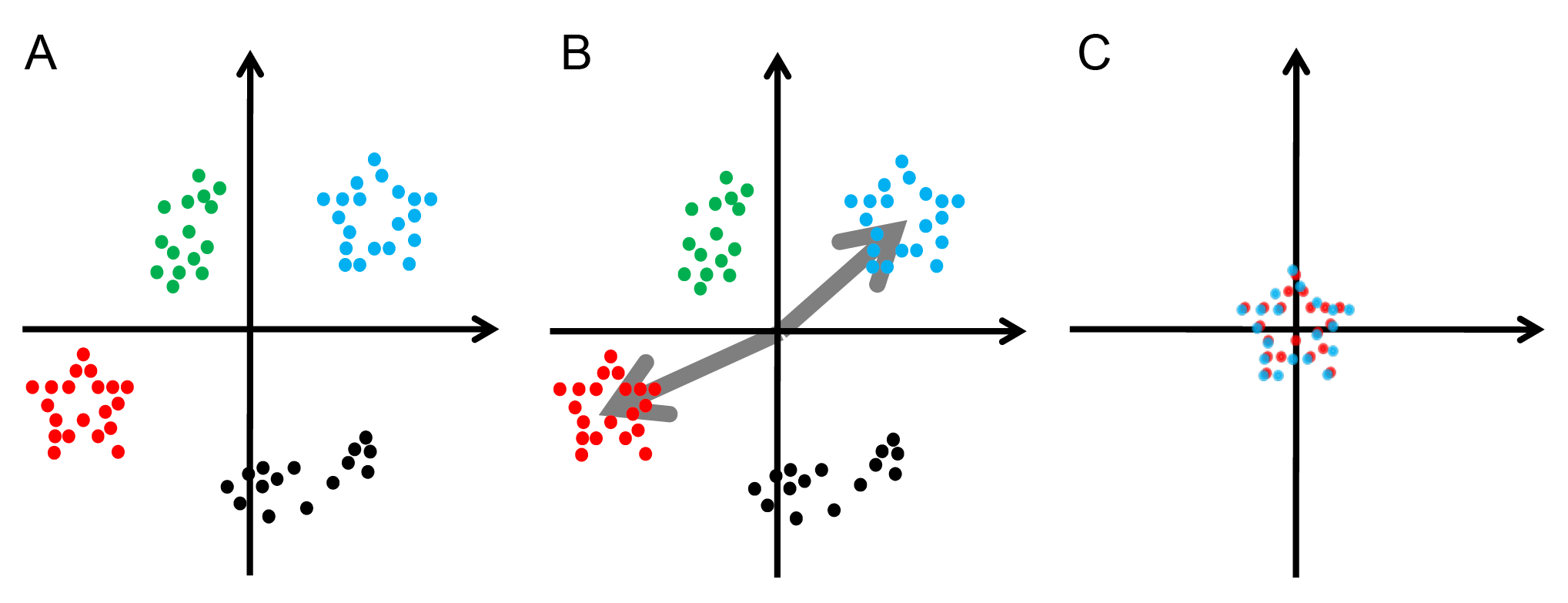

Supplement: Figure S1 — Analysis of residuals from the first round of LLE in RepEdLEGG allows detection of minor similarity (Conceptual diagram). For the sake of visualization, log-transformed expression level ratio values of two genes (i.e., two dimensions) are plotted for 70 mutant plants, which correspond to the data points. (A) Major similarities among the data points can be identified as clusters in the global space. Four clusters are indicated by different colors of the data points. (B) For each of the red and blue clusters, major components (thick gray arrows) are identified. This can be achieved by the first round of LLE. (C) Once the major components are subtracted from the red and blue clusters (i.e., residuals), minor similarities (the star-like shapes of the clusters) can be identified. Note that since it is impossible to represent events in a high-dimensional space accurately in a two-dimensional space, this figure is by no means an accurate representation of the RepEdLEGG procedure. Instead, the purpose of this figure is to illustrate the idea that analysis of residuals allows detection of minor similarities in expression profile data, which correspond to weak regulatory relationships among the genes corresponding to the mutations. (4.81 MB TIF) [file ppat.1001011.s001.tif]

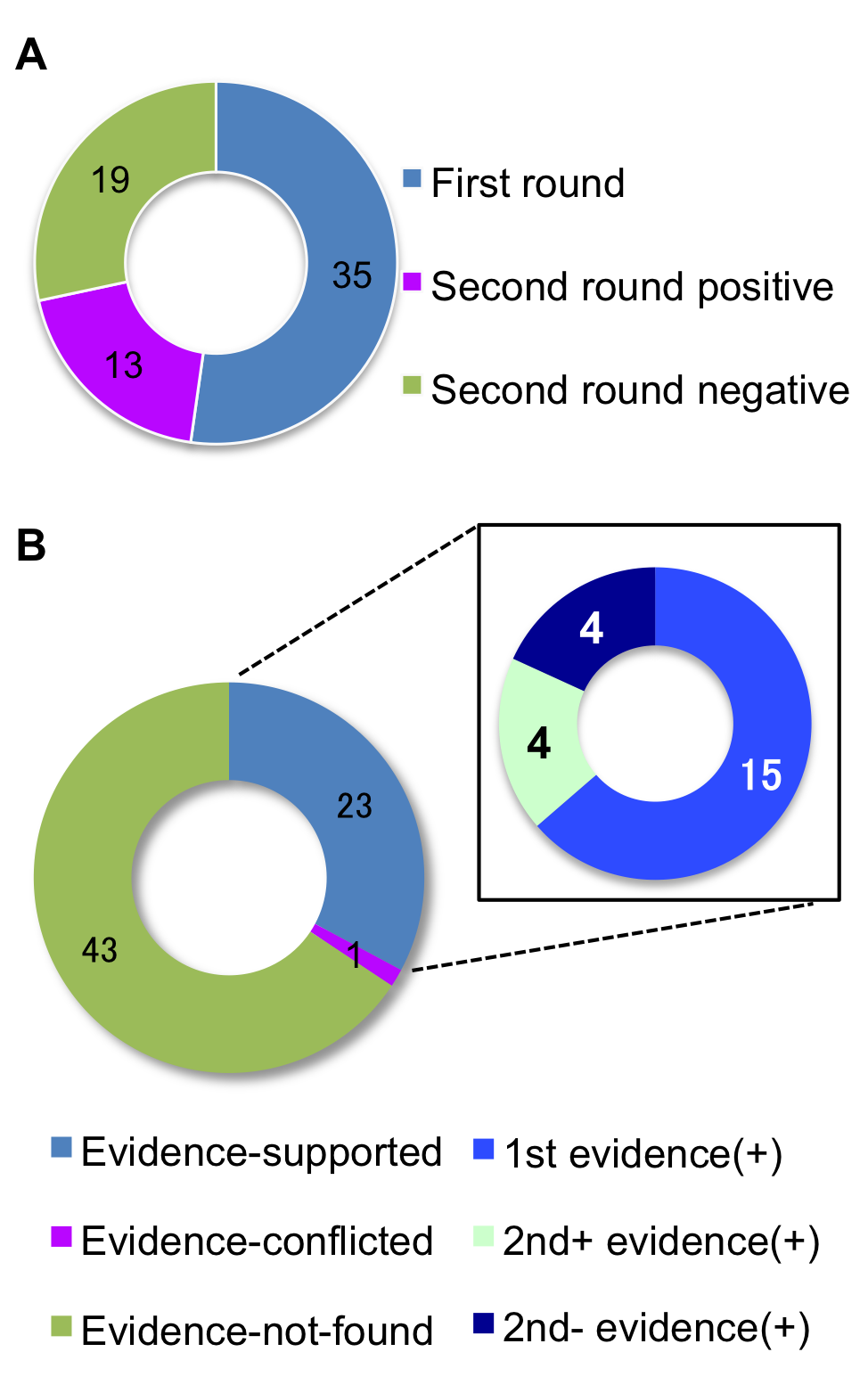

Supplement: Figure S2 — Categories of links inferred by RepEdLEGG. (A) Links inferred by the RepEdLEGG procedure are classified into those inferred in the first and second rounds of the LLE procedure. The links inferred in the second round are further divided into positive and negative links. (B) Links with supporting, conflicting or no evidence. The chart in the inset indicates the proportion of evidence-supported links inferred in the first and second rounds. 1st evidence (+), evidence-supported links inferred in the first round of LLE; 2nd+ evidence (+), evidence-supported links for positive regulatory relationships inferred in the second round of LLE; 2nd- evidence (+), evidence-supported links for negative regulatory relationships inferred in the second round of LLE. Supporting evidence is listed in Table S3. (0.24 MB TIF) [file ppat.1001011.s002.tif]

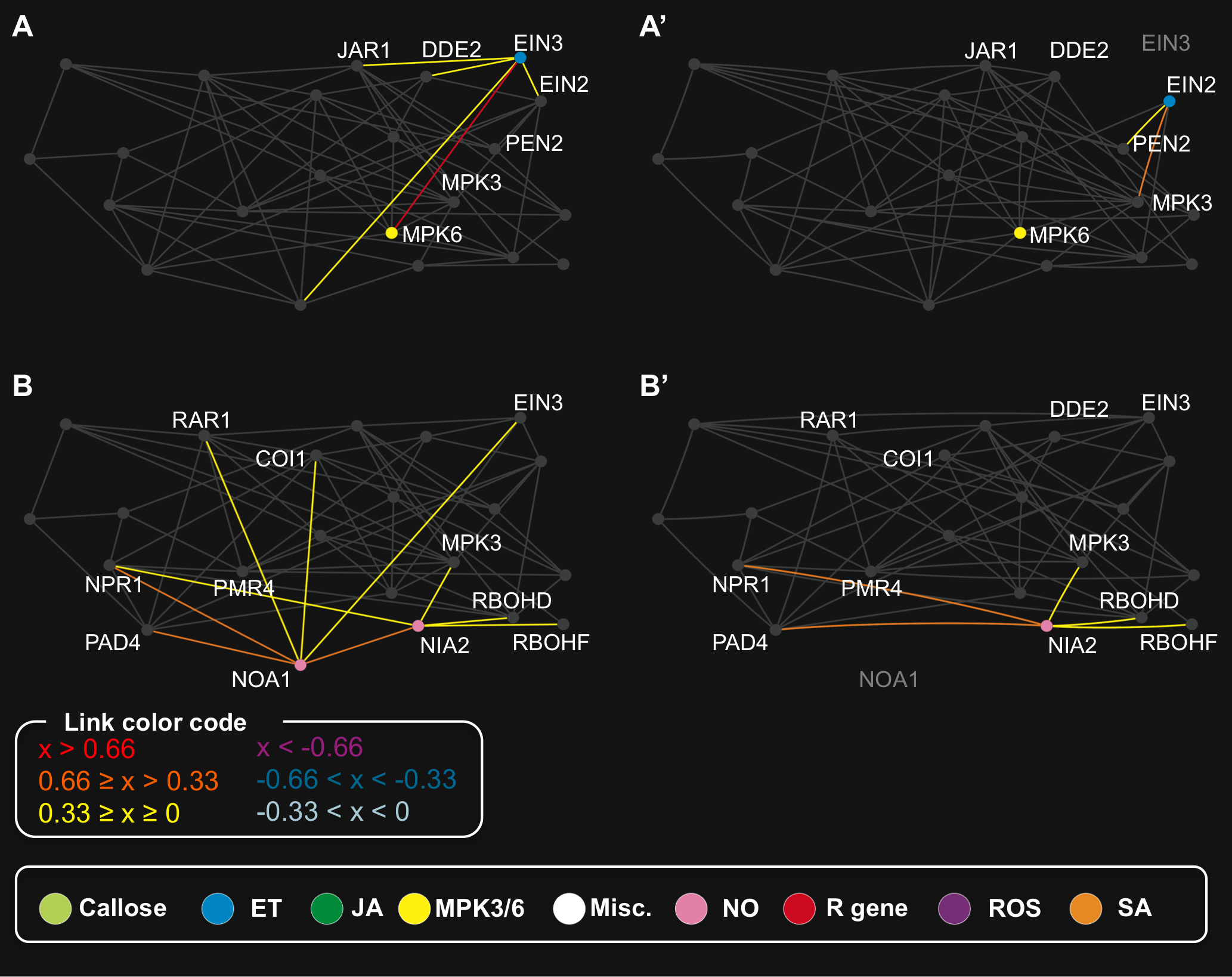

Supplement: Figure S3 — Analyses of the link specificities. (A and A′) The link between EIN3 and MPK6 is not ET signaling-dependent. To test specificity of the link between EIN3 and MPK6, the ein3 profile was removed from the data set, and the RepEdLEGG analysis was performed. A, The links involving EIN3 are highlighted in the full model (Figure 2C). A′, The links involving EIN2 are highlighted in the model with EIN3 removed. (B and B′) The NO sector has positive relationships with the SA sector. To analyze the specificity of links between the NO sector components (NOA1 and NIA2) and the SA sector components, the RepEdLEGG analysis was performed with the data set with the noa1 profile removed. B, The links involving the NO signaling components are highlighted in the full model (Figure 2C). B′, The links involving NIA2 are highlighted in the model without NOA1. Links inferred 17 times in the LOO cross-validation results were considered significant when a data set with one mutant profile removed is used. Note that a link can be inferred 19 times at maximum when one component is removed. The color codes of the links were determined based on coefficients associated with the links. The color codes for the vertices at the bottom of the figure show the signaling sector assignments of the genes corresponding to the mutations. (1.01 MB TIF) [file ppat.1001011.s003.tif]

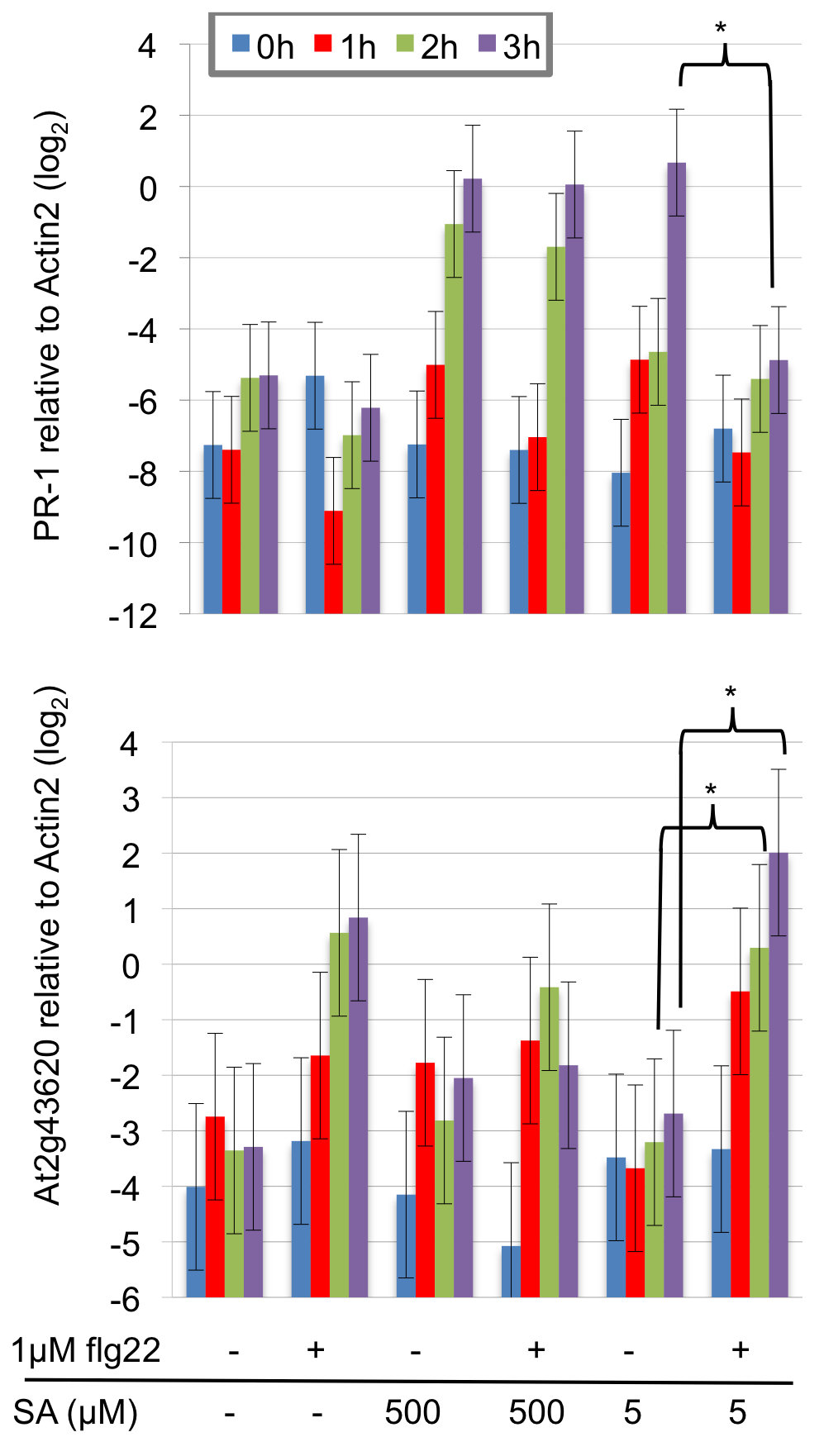

Supplement: Figure S4 — Time-course analysis of mutual inhibition between the EMT and SA sectors. The mRNA levels of the PR-1 and putative chitinase (At2g43620) genes were measured by qRT-PCR and used as proxies of the SA and EMT sector activities, respectively. The Actin 2 mRNA level was used to normalize the mRNA measurements. Ten-day old seedlings were treated with indicated concentrations of SA and/or flg22 and harvested for mRNA measurements at the indicated times. *, p<0.05 for the indicated comparisons. (0.32 MB TIF) [file ppat.1001011.s004.tif]

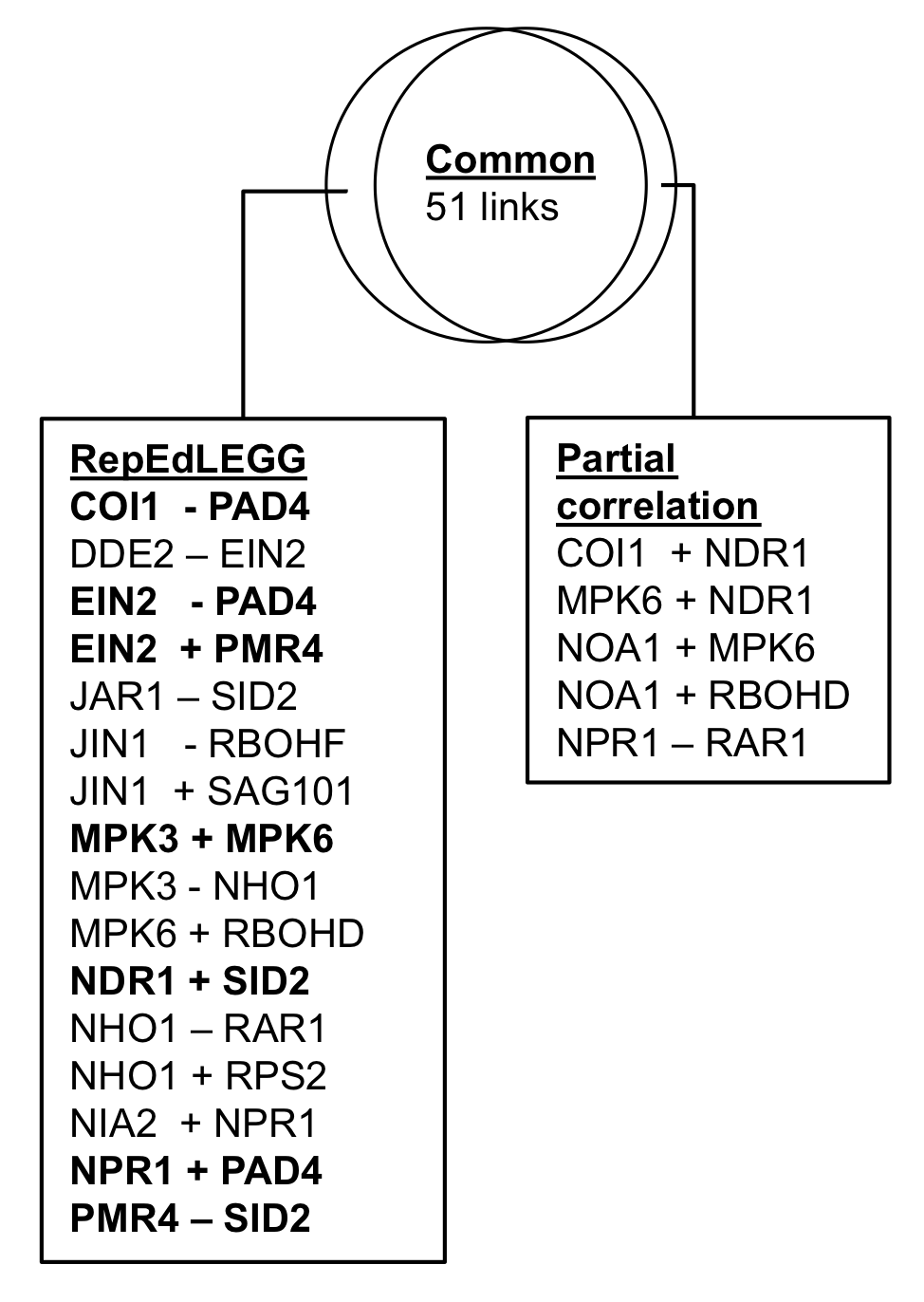

Supplement: Figure S5 — Comparison of RepEdlEGG with partial correlation. The expression ratios between mutants and Col-0 wild-type (22 genotypes×480 genes) were analyzed using RepEdLEGG and partial correlation with LOO cross-validation. Links inferred 18 times in the LOO results were considered significant. (0.19 MB TIF) [file ppat.1001011.s005.tif]
